# Bioinspired Multifunctional Flexible C‐SiC Fibrous Aerogel for Superior Electromagnetic Interference Shielding Under Extreme Environments

**DOI:** 10.1002/advs.76343

**Published:** 2026-07-07

**Authors:** Tianyue Yang, Yan Shen, Zhongqian Zhao, Xue Zhou, Qianji Chen, Xujing Wang, Chuchu Zheng, Zhaoxiang Lu, Yangzhong Zhao, Yanzi Gou

**Affiliations:** ^1^ Science and Technology on Advanced Ceramic Fibers and Composites Laboratory College of Aerospace Science and Engineering National University of Defense Technology Changsha China

**Keywords:** C‐SiC fibrous aerogels, electromagnetic interference shielding, multifunctional, precursor, thermal conductivity

## Abstract

The flexible, lightweight, and high‐temperature‐resistant aerogels with both thermal insulation and electromagnetic shielding functions are urgently required in many high‐tech fields. Although carbon‐based aerogels are promising, they are not satisfactory due to complex manufacturing processes and poor oxidation resistance (<500°C). Therefore, it is still a great challenge to design materials with processing advantages and multiple functions. Drawing inspiration from the unique structure of momordica charantia, this work developed a biomimetic C‐SiC fibrous aerogel by employing low‐cost polysiloxane and polyacrylonitrile as precursor materials. The C‐SiC fiber exhibited special surface morphology with protrusions and hierarchical pores, as well as a loose core after a fabrication procedure involving centrifugal spinning and high‐temperature sintering processes. The momordica charantia‐like structure endowed the flexible C‐SiC fibrous aerogel with ultralow density of 3.9 mg cm^−3^, exceptionally low thermal conductivity (9.12 mW m^−1^ K^−1^), and an ultrahigh specific electromagnetic shielding effectiveness (346 828.6 dB cm^2^ g^−1^). Besides compressibility and resilience, the aerogel exhibited outstanding acid‐alkali corrosion resistance, ultrahigh‐temperature stability (2000°C), and relatively good oxidation resistance (1000°C). This work delivered a scalable, cost‐effective biomimetic strategy for fabricating high‐performance fibrous aerogels as next‐generation structure‐function integrated materials, with potential applications in aerospace thermal protection and related high‐temperature environments.

## Graphical abstract

This schematic presents a biomimetic C‐SiC fibrous aerogel inspired by the special structure of momordica charantia. It exhibits outstanding thermal insulation and ultrahigh specific electromagnetic shielding performance, together with excellent high‐temperature stability and mechanical flexibility, showing great promise for aerospace applications under various extreme environments.

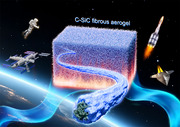

## 1 Introduction

With the increasing demand for multiple protection in extreme environments, especially in fields such as aerospace, equipment, and precision instruments, there is an urgent need to develop materials with both thermal insulation and electromagnetic interference (EMI) shielding properties [1, 2, 3, 4]. In particular, given the harsh conditions of outer space characterized by significant temperature fluctuations (−150°C to 150°C) and intense radiation, extravehicular spacesuits with functions of thermal control and EMI shielding have become indispensable protective gear for astronauts conducting activities outside the space station [5, 6, 7]. Moreover, the temperature can surge up to 1000°C for the sensing components of the exhaust nozzle of aero‐engines under an oxygen‐containing environment, where the integrated demand for efficient thermal insulation and EMI shielding is also critical [8, 9, 10, 11, 12]. Therefore, the development of flexible, lightweight, and low‐cost materials with both EMI shielding and thermal insulation properties, especially those adaptable to conditions of wide temperature ranges or high‐temperature aerobic environments, is a major requirement in the aerospace field [13, 14, 15, 16].

Carbon‐based aerogels are promising for application in the electromagnetic shielding field within wide temperature ranges. Liang et al. fabricated MXene aerogel/wood‐derived porous carbon composites [17], which delivered an optimal EMI shielding effectiveness (SE) of 71.3 dB with a low density of 197 mg cm^−3^. Han et al. prepared reduced graphene oxide–carbon nanotube‐vertical edge‐rich graphene (rGO‐CNT‐VG) aerogels by chemical vapor deposition [18], exhibiting a thermal conductivity of 2460 mW m^−1^ K^−1^ and an outstanding EMI shielding effectiveness of 57 dB. Qiao et al., obtained polyimide/carbon nanotube CNT aerogels by the freeze‐drying method [19], yielding an EMI SE of 71 dB and a specific efficiency of 6470 dB cm^2^ g^−1^. Chen et al. fabricated ultralight, conductive, and multi‐scale core‐skin structured carbon aerogels via freeze‐drying and heat treatment of polydopamine nanofibers [20]. In the X‐band, with a density of 3.11 mg cm^−3^ and a thickness of 2 mm, its specific shielding effectiveness (SSE/t) value reached 47910 dB cm^2^ g^−1^. Ma et al. fabricated PG@PyC/AZ91D composites via a 3D skeleton preconstruction‐infiltration method, realizing an EMI shielding effectiveness of 76.70 dB at 3 mm thickness [21]. Ghaffarkhah et al. developed Ti_3_C_2_T*_x_*/GO cryogels, achieving exceptional EMI shielding effectiveness (44.7–69.2 dB), specific EMI shielding effectiveness (33 000–50 000 dB cm^2^ g^−1^), and ultra‐low density of 3–7 mg cm^−3^ [22]. Thereof, carbon‐based aerogels have already been developed for electromagnetic shielding [23, 24, 25, 26, 27]. However, it should not be overlooked that the present carbon‐based aerogels are plagued by several inherent drawbacks, including intricate preparation processes, high production costs, and impediments to large‐scale manufacturing [28, 29]. More critically, they are inherently susceptible to oxidation in oxygen‐containing atmosphere (<500°C), which severely curtails their practical applications in the realm of high‐temperature aerobic environment [30]. Fortunately, this drawback can be effectively mitigated by incorporating a SiC phase into carbon‐based materials. The rationale lies in the fact that SiC boasts exceptional high‐temperature stability and oxidation resistance [31, 32, 33, 34, 35]. Furthermore, centrifugal spinning technology offers distinct advantages for large‐scale production and integrated aerogel molding. Guided by bionic design principles, it is highly feasible to realize hierarchical structural engineering using low‐cost raw materials, thereby endowing the resultant aerogels with superior thermal insulation and EMI shielding capabilities.

In this work, by using inexpensive precursors consisting of polysiloxane and polyacrylonitrile, we successfully prepared a momordica charantia‐like C‐SiC fibrous aerogel through a precursor‐derived procedure including centrifugal spinning and high‐temperature sintering processes. Inspired by the special surface and core structure of Momordica charantia, the surface with granular protrusions, grooves, and pores was constructed on the C‐SiC fiber to lengthen the heat transfer path, and the simultaneously formed loose core of the fiber was employed to reduce the heat conduction efficiency, which jointly achieved excellent heat insulation performance. Furthermore, by utilizing the porous scattering on the single fiber surface and the continuous conductive path formed by the 3D network of multiple fibers, the electromagnetic shielding function was also realized through electromagnetic wave reflection, absorption, and multiple scattering. Additionally, the loose core of the momordica charantia‐like fiber was also designed to effectively reduce the weight to achieve the goal of lightweight. Therefore, the ultralight (3.9 mg cm^−3^) C‐SiC fibrous aerogel exhibited an extremely low thermal conductivity (9.12 mW m^−1^ K^−1^), as well as excellent EMI shielding performance with a maximum electromagnetic shielding effectiveness (SE) of 53.1 dB and SSE/t up to 346 828.6 dB cm^2^ g^−1^. Importantly, the C‐SiC fibrous aerogel also showed excellent comprehensive performance including high‐temperature resistance (2000°C for 1 h), oxidation resistance (1000°C), corrosion resistance to acids and alkalis (for 7 days in 1 M HCl or 6 M NaOH), and mechanical properties (ε = 60%, 85 cycles). The processing advantages and synergistic structural‐functional properties of the C‐SiC fibrous aerogel in this work not only provide a new paradigm for optimizing the performance of biomimetic materials, but also offer a low‐cost and scalable manufacturing prototype for effective thermal insulation and electromagnetic interference shielding in extreme environments.

## 2 Results and Discussion

### 2.1 Fabrication of the C‐SiC Fibrous Aerogel

**FIGURE 1:**
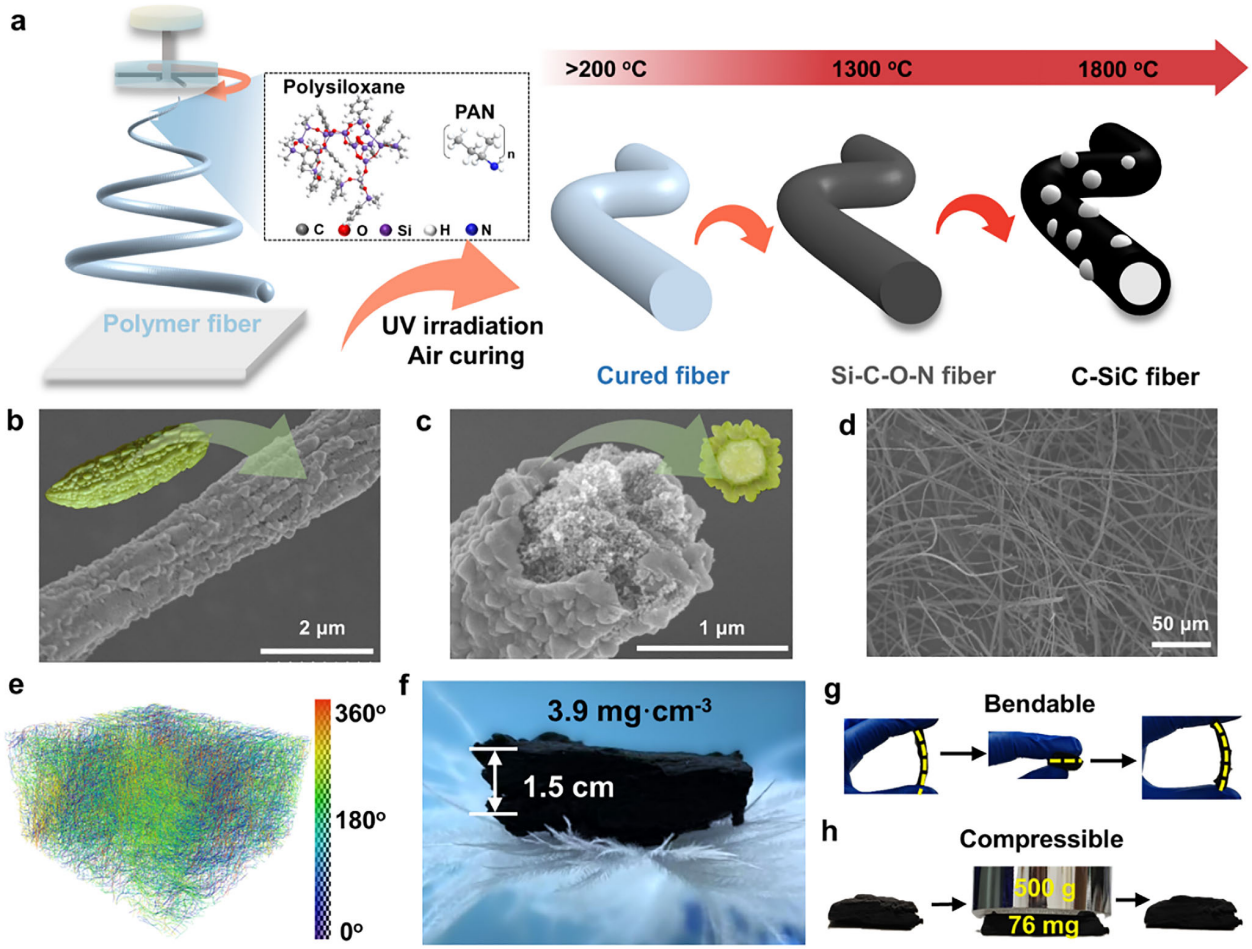
Fabrication of the C‐SiC fibrous aerogels. (a) Schematic diagram of the fabrication process for the C‐SiC fibrous aerogels. (b) SEM image of the individual fiber surface. (c) SEM image of the individual fiber cross‐section. (d) SEM image of the C‐SiC fibrous aerogels. (e) 3D reconstructed CT image of the C‐SiC fibrous aerogel (with different colours indicating the orientation of the fibers). (f) Optical image of the C‐SiC fibrous aerogel standing on the tip of a feather. (g) Optical images of the C‐SiC fibrous aerogel before and after bending. (h) Optical images of the C‐SiC fibrous aerogel before and after compression.

The C‐SiC fibrous aerogels were prepared via a precursor‐derived method (Figure 1a). Firstly, polysiloxane and PAN were used as the Si source and C source, respectively, to obtain spinning solutions with different ratios. After centrifugal spinning, polymer fibrous aerogels were obtained (Figures S1–S5). Further, they were transformed to cured fibrous aerogels by ultraviolet (UV) irradiation and subsequent cross‐linking in an air atmosphere, which enabled the fiber morphology to be maintained during the subsequent pyrolysis process (Figures S6–S8). Next, the cured fibrous aerogels were treated at 1300°C for 1 h to obtain inorganic Si‐C‐O‐N fibrous aerogels (Figures S9–S13). Finally, the Si‐C‐O‐N fibrous aerogels were sintered at 1800°C in an inert atmosphere, resulting in C‐SiC‐X fibrous aerogels (where C‐SiC‐X represented the different C‐SiC aerogels obtained by varying the ratio of polysiloxanes to PAN). By controlling the overflow of gas molecules during the sintering stage, granular protrusions were formed on the fiber surface with the appearance of grooves, showing a morphology closely resembling that of Momordica charantia (Figure 1b). Cross‐sectional analysis of the fiber revealed a loose core contrasted by a relatively dense outer shell, a configuration analogous to the internal anatomy of momordica charantia (Figure 1c). Such structural characteristics contributed to the reduction in the overall weight of the aerogel materials. A scanning electron microscopy (SEM) image revealed that the fibers inside the C‐SiC fibrous aerogel were arranged in a random orientation (Figure 1d). This observation was further corroborated by three‐dimensional (3D) computed tomography (CT) scans of the C‐SiC fibrous aerogel, which revealed a porosity of 95.5% (Figure 1e). The C‐SiC fibrous aerogel rested on an ostrich feather, illustrating their exceptionally low density (3.9 mg cm^−3^) (Figure 1f). Notably, the aerogel demonstrated the ability to recover its original shape after deformation by a 180° bending (Figure 1g). Furthermore, after being compressed by a load approximately 6500 times its own weight, the aerogel exhibited compressive resilience and rebound behavior (Figure 1h).

### 2.2 Transformation of the Si‐C‐O‐N to the C‐SiC Fibrous Aerogel

**FIGURE 2:**
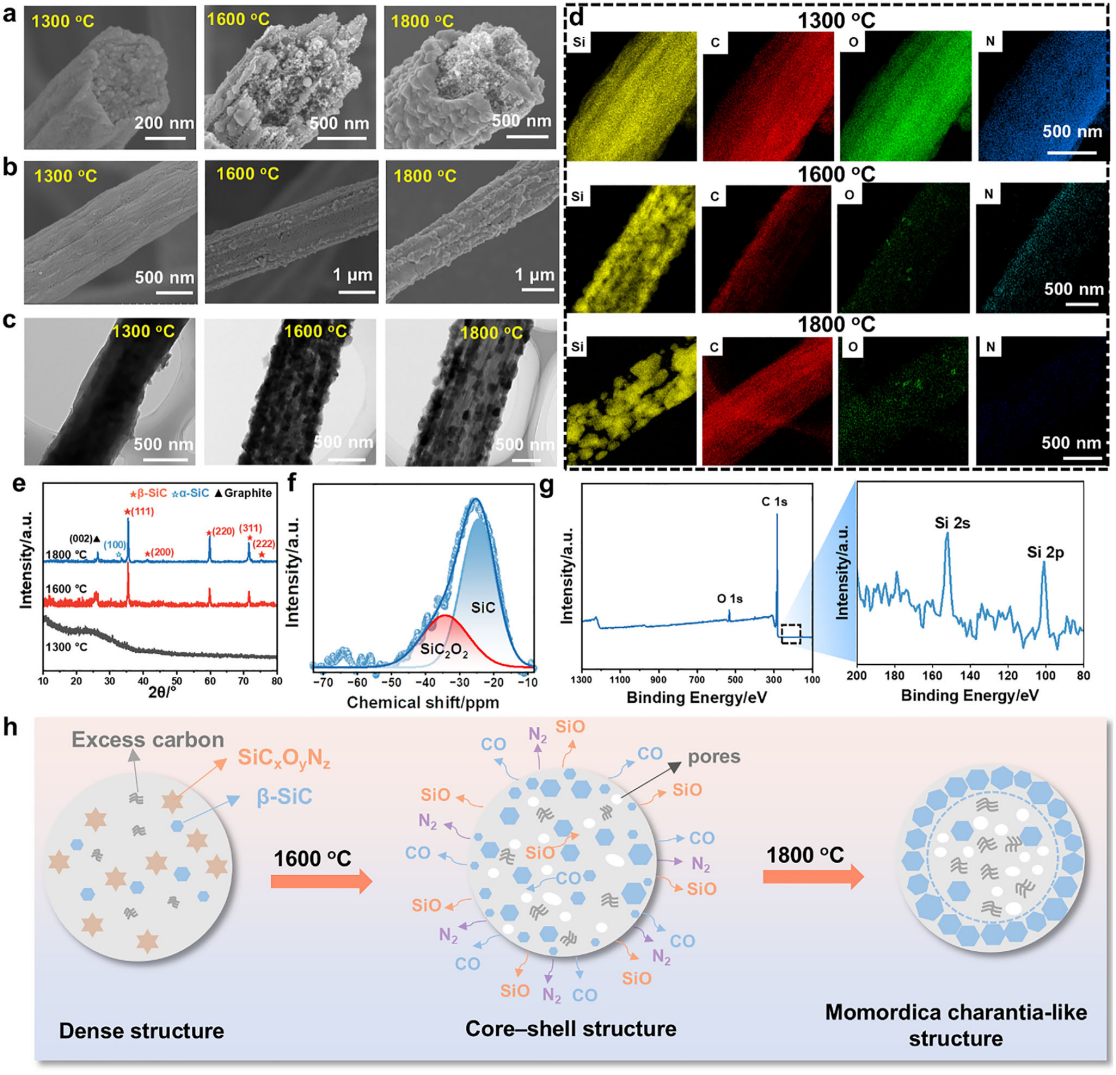
Transformation of the Si‐C‐O‐N to the C‐SiC fibrous aerogel. (a) SEM images of the fiber cross‐sections obtained at different temperatures. (b) SEM images of the fiber surface obtained at different temperatures. (c) TEM images of the fibers obtained at different temperatures. (d) TEM‐EDS images of the fibers obtained at different temperatures. (e) XRD pattern of the fibrous aerogels obtained at different temperatures. (f) ^29^Si MAS NMR spectrum of the C‐SiC fibrous aerogel obtained at 1800°C. (g) XPS spectra of the C‐SiC fibrous aerogels obtained at 1800°C. (h) Schematic diagram of transformation mechanism at high temperatures.

The transformation of dense Si‐C‐O‐N to momordica charantia‐like C‐SiC fibrous aerogels was investigated in detail. The Si‐C‐O‐N fibrous aerogels obtained at 1300°C were further heated to 1600°C and 1800°C, respectively, to elaborate on the changes in the structure and composition of fibers during the high‐temperature evolution process. It could be seen that the fiber underwent transformation from a solid configuration to a core–shell architecture (Figure 2a; Figures S14 and S15). Concurrently, the fiber surface morphology altered from a relatively smooth texture to a granular protrusion pattern (Figure 2b). Transmission electron microscopy (TEM) images revealed that with increasing temperature, there was an enhancement in the SiC crystalline phase. The presence of a momordica charantia‐like morphology was also corroborated by the TEM observations (Figure 2c; Figures S16 and S17). Energy‐dispersive spectroscopy (EDS) analysis indicated that the fiber obtained at 1300°C consisted of four elements: silicon (Si), carbon (C), nitrogen (N), and oxygen (O). However, the contents of nitrogen and oxygen progressively decreased at 1600°C. Upon high‐temperature sintering at 1800°C, only silicon and carbon remained (Figure 2d). This observation was validated by quantitative elemental analysis of the aerogels. After sintering at 1800°C, the oxygen content reduced from 18.64 wt.% of the Si‐C‐O‐N to only 0.31 wt.% of the C‐SiC fibrous aerogels, whereas the nitrogen content decreased from 10.08 to 0.32 wt.% (Table S1). It was observed that the C‐SiC fibrous aerogels obtained at 1800°C were carbon‐rich, with C/Si atomic ratio descending from 9.59 to 3.37 as the feeding ratio of polysiloxane to PAN increased from 0.5 to 3 (Table S2).

The X‐ray diffraction (XRD) patterns (Figure 2e and Figure S18) indicated an amorphous structure of the Si‐C‐O‐N fibrous aerogels obtained at 1300°C with a broad scattering peak centered around 25.0°. Upon increasing the temperature to 1600°C, distinct diffraction peaks at 35.6°, 60.0°, and 71.8°, corresponding to the (111), (200), and (311) crystal planes of β‐SiC, respectively, confirmed the presence of β‐SiC crystalline phase in the fibrous aerogel. A week peak attributed to the (100) plane of α‐SiC was also detected. Additionally, a pronounced diffraction peak at 2θ = 26.2° was observed, which could be attributed to the (002) plane of graphite. The grain size of β‐SiC was determined using the Scherrer equation, and the calculated values were summarized in Table S3. It could be seen that sintering at 1800°C resulted in an increased grain size of the β‐SiC. It was confirmed by ^29^Si MAS NMR spectrum (Figure 2f) that Si in the fiber existed as β‐SiC and SiC_2_O_2_, according to the major peak at −24 ppm and the weak peak at −34 ppm, respectively [36]. Furthermore, X‐ray photoelectron spectroscopy (XPS) demonstrated the presence of C, O, and Si elements (Figure 2g) [36, 37]. Raman spectroscopy results indicated that the degree of graphitization exhibited an increasing trend with elevated temperatures (Figures S19 and S20).

By heating the Si‐C‐O‐N fibrous aerogels at temperature up to 1800°C, the reactions depicted in Equations (1) and (2) occurred [38, 39, 40]. The gaseous species of SiO, CO, and N_2_, produced by the decomposition of the SiC_x_O_y_N_z_ phase of the fibers (Equation 1), tended to migrate outward. The outward diffusion of SiO was hindered upon reaching the carbon layer near the surface, which promoted the carbothermal reduction reaction, resulting in the formation of SiC (Equation 2). Consequently, the SiC grains formed in this region exhibited large grain size and strong intergranular bonding, thereby constituting the outer shell of the momordica charantia‐like fiber. In contrast, the continued outward migration of SiO from the core led to an accumulation of carbon, fostering the formation of a carbon‐rich and loose core.

(1) $$\mathrm{Si}C_{x}O_{y}N_{z}\left(s\right)\to \mathrm{SiC}\left(s\right)+C\left(s\right)+\mathrm{SiO}\left(g\right)+\mathrm{CO}\left(g\right)+N_{2}\left(g\right)$$

(2) $$\mathrm{SiO}\left(g\right)+C\left(s\right)\to \mathrm{SiC}\left(s\right)+\mathrm{CO}\left(g\right)$$

As elucidated by Figure 2h, the Si‐C‐O‐N fibrous aerogel obtained at 1300°C primarily consisted of an amorphous SiC_x_O_y_N_z_ phase, with a considerable presence of impurity elements such as oxygen and nitrogen. Upon being heated at 1600°C, there was a noticeable reduction in the content of both impurities. Additionally, pores formed in the interior of the fibers, accompanied by the appearance of the core–shell structure. By sintering at 1800°C, the as‐prepared C‐SiC fibrous aerogel exhibited increased grain size and densification of the SiC shell, resulting in the whole momordica charantia‐like structure. The evolution from dense Si‐C‐O‐N fibers to hierarchical core‐shell structures is expected to promote the collaborative optimization of the thermal insulation and EMI shielding properties of C‐SiC fibrous aerogels through the biomimetic design.

### 2.3 Mechanical and Chemical Properties

**FIGURE 3:**
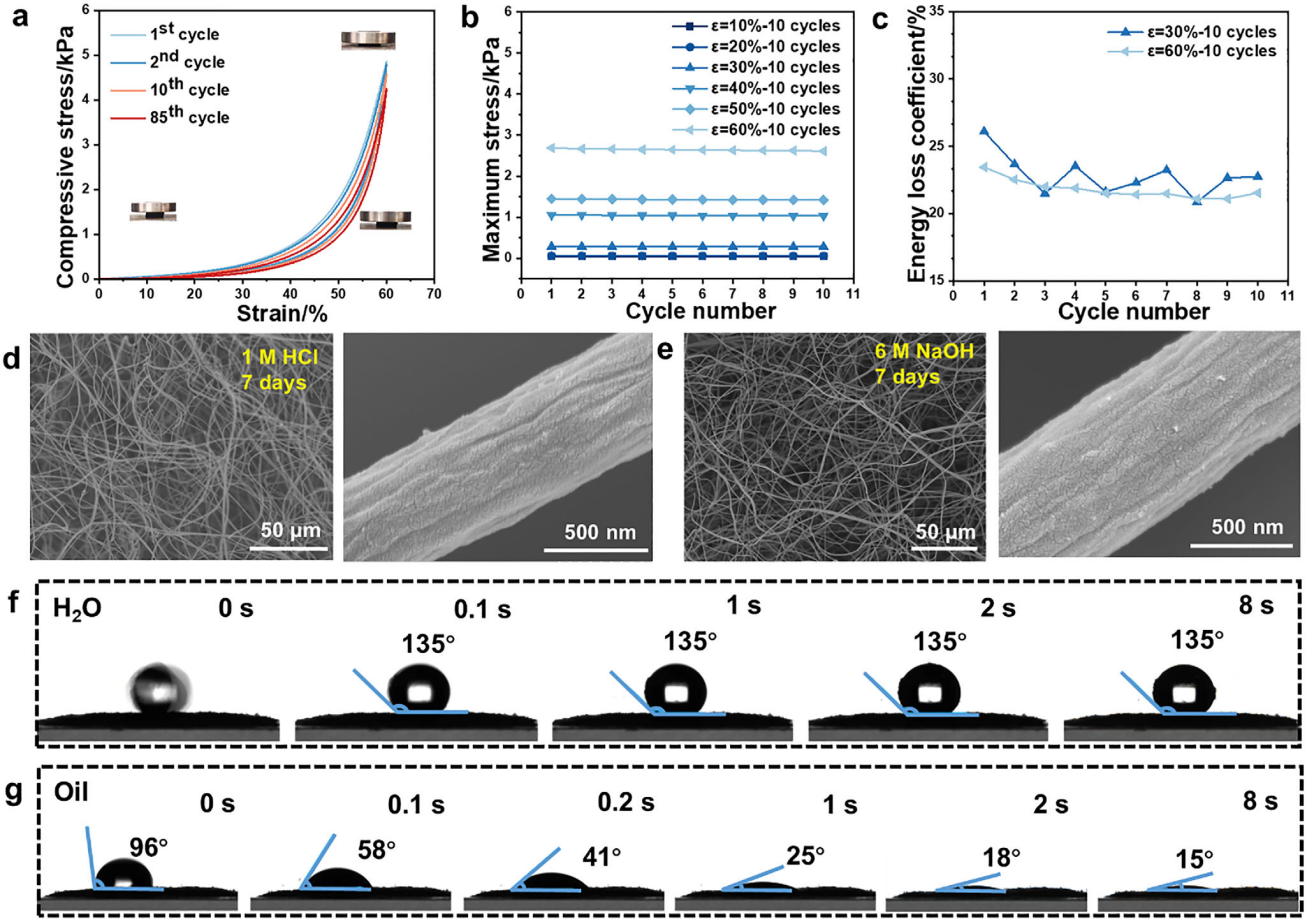
Mechanical and chemical properties of the C‐SiC fibrous aerogel. (a) Stress‐strain curves of the C‐SiC fibrous aerogel. (b) Maximum compressive stresses and (c) Energy loss coefficients for 10 cycles at ε = 30% and 60%. (d) SEM images of the C‐SiC fibrous aerogel after being held in 1 M HCl for 7 days. (e) SEM images of the C‐SiC fibrous aerogel after being held in 6 M NaOH for 7 days. (f) Water contact angle and (g) Oil contact angle of the C‐SiC fibrous aerogel.

The C‐SiC fibrous aerogels possessed good compressibility, corrosion resistance, and hydrophobicity. As shown by the compression cycle tests, the C‐SiC fibrous aerogel maintained compression‐rebound behavior at a strain of ε = 60% to 85 cycles (Figure 3a). Typically, the fibrous aerogel exhibited a linear elastic region at low strain values (ε < 10%), which transitioned to a plastic deformation region at moderate strain (10% < ε < 65%). The material maintained a stable load‐bearing capacity under compression strains ranging of 10%–60% for 10 cycles (Figure 3b). The energy loss coefficient generally displayed minor fluctuations, stabilizing around 25% (Figure 3c). The tensile strength of the C‐SiC fibrous aerogel was 97.9 kPa (Figure S21). Notably, the C‐SiC fibrous aerogel preserved its compression‐rebound properties under liquid nitrogen exposure (−196°C) or heat treatment by using an alcohol lamp (∼800°C) (Figures S22 and S23).

The C‐SiC fibrous aerogels exhibited notable resistance to chemical corrosion. Specifically, it was revealed that after being held in 1 M hydrochloric acid (Figure 3d and Figure S24) and 6 M sodium hydroxide (Figure 3e and Figure S25) solutions, respectively, at ambient temperature for a duration of seven days, the C‐SiC fibrous aerogel remained a continuous and uniform morphology without any etching. Moreover, the C‐SiC fibrous aerogel also maintained a certain compression‐rebound performance (Figure S26). To further investigate the surface wettability characteristics of the C‐SiC fibrous aerogel, contact angle measurements were performed using water and oil as test liquids. The C‐SiC fibrous aerogel achieved a stable contact angle of 135° with water within 100 ms, indicating pronounced hydrophobicity (Figure 3f). Dynamic observations showed that the contact angle between the aerogel and oil decreased from 96° to 15°, culminating in complete oil absorption (Figure 3g). These wettability behaviors could be attributed to high porosity, substantial surface roughness, and large specific surface area of the momordica charantia‐like structure [41]. Collectively, these features confer a hydrophobic‐oleophilic surface to the C‐SiC fibrous aerogel. The combination of these properties suggests great potential for the application of such fibrous aerogels in complex environmental conditions.

### 2.4 High‐Temperature Resistance and Thermal Insulation Performance

**FIGURE 4:**
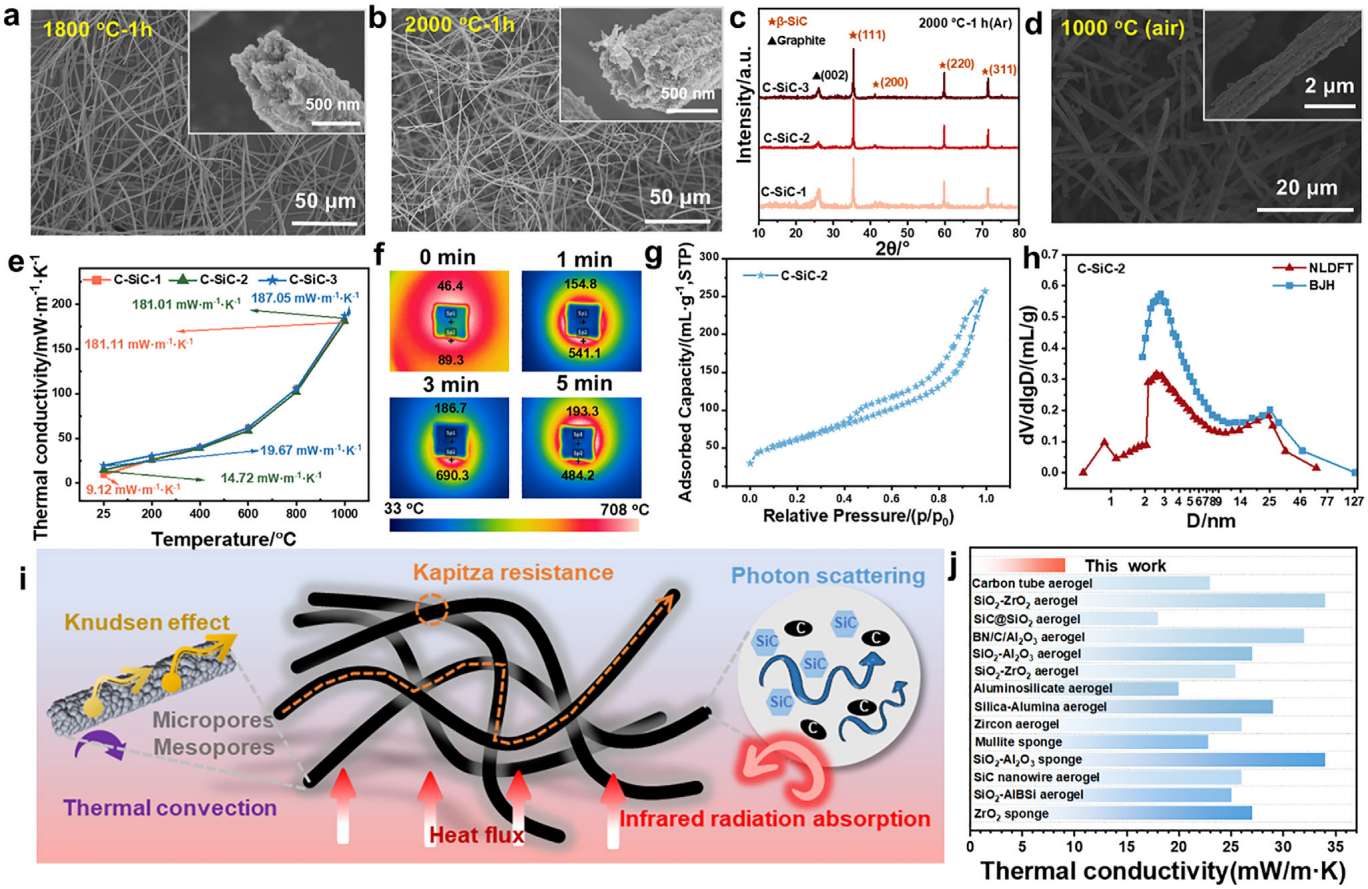
High‐temperature resistance and thermal insulation performance of the C‐SiC fibrous aerogel. (a) SEM images of the C‐SiC fibrous aerogels after heat treatment at 1800°C for 1 h. (b) SEM images of the C‐SiC fibrous aerogels after heat treatment at 2000°C for 1 h. (c) XRD pattern of the C‐SiC fibrous aerogels after heat treatment at 2000°C for 1 h. (d) SEM images of the C‐SiC fibrous aerogel after oxidation at 1000°C for 10 min. (e) Thermal conductivity of the C‐SiC fibrous aerogels at different temperatures. (f) Infrared images of the C‐SiC fibrous aerogels being heated by a butane torch. (g) N_2_ adsorption‐desorption isotherms of the C‐SiC fibrous aerogels. (h) Differential pore volume vs. logarithmic pore size distribution curves of the C‐SiC fibrous aerogels obtained by NLDFT and BJH (adsorption) methods. (i) Schematic diagram of the thermal insulation mechanism of the C‐SiC fibrous aerogels. (j) Comparison chart of the thermal conductivity of the C‐SiC fibrous aerogels in this work and those reported in the literature.

The C‐SiC fibrous aerogels exhibited excellent high‐temperature resistance and oxidation resistance. After being treated at 1800°C for 1 h under a flowing argon atmosphere, the mass retention of the C‐SiC fibrous aerogel was approximately 99%, with the fiber morphology remaining unchanged (Figure 4a and Figure S27). Furthermore, after heat treatment at 1800°C for 5 h, the C‐SiC fibrous aerogel maintained its composition and structural stability (Figures S28 and S29). Significantly, even after treatment at 2000°C for 1 h in Ar atmosphere, the fibrous aerogel still preserved its structural integrity (Figure 4b and Figure S30) with the same crystalline components (Figure 4c). Interestingly, following heat treatment at 2000°C for 1 h under vacuum, the C‐SiC fibrous aerogel also maintained its structural integrity (Figure S31). For comparative purpose, commercially available carbon fibrous aerogels and the C‐SiC fibrous aerogels were subjected to oxidation at 700°C for 1 h. The carbon aerogels almost disappeared, with only tiny residue left (Figures S32–S34). In contrast, the C‐SiC fibrous aerogels retained the fiber structure, underscoring much better oxidation resistance (Figure S35). Importantly, after oxidation at 1000°C in air for 10 min, the C‐SiC fibrous aerogel largely retained its fibrous morphology, indicating good structural stability against oxidation (Figure 4d and Figure S36).

The C‐SiC fibrous aerogels showed superior thermal insulation performance. Thermal conductivity of the C‐SiC fibrous aerogels was measured from room temperature to 1000°C (Figure 4e). At room temperature, the thermal conductivity was extremely low (9.12 mW m^−1^ K^−1^). Furthermore, the thermal conductivity at 1000°C remained very low, approximately 181.11 mW m^−1^ K^−1^. To gain a deeper insight into the thermal insulation properties, the C‐SiC fibrous aerogel with approximately 1 cm in thickness was subjected to heating by the butane torch flame, which was supported on an asbestos mesh (Figure 4f, Figure S37, and Movie S1). The temperature measured at the heated end of the C‐SiC fibrous aerogel exceeded 820°C. With prolonged time, the temperature at the unheated end rose from an initial ambient temperature of 16.9°C to approximately 195°C, subsequently stabilizing. The resulting temperature gradient between the hot and cold ends surpassed 600°C, demonstrating superior thermal insulation capabilities.

The porous architecture is a critical factor influencing the thermal insulation properties of the C‐SiC fibrous aerogels [35, 42]. By examining the N_2_ adsorption‐desorption isotherms, essential characteristics including the pore structure, specific surface area, and pore size distribution of the C‐SiC fibers inside the aerogels were comprehensively evaluated (Figure 4g and Figure S38). The adsorption curve corresponded to a type‐II adsorption isotherm, while the desorption curve exhibited an H3‐type hysteresis loop [43, 44, 45]. The N_2_ adsorption isotherm could be delineated into three distinct regions with relative pressures (p/p_0_) below 0.02, 0.02 to 0.89, and above 0.89, respectively. These three regions corresponded to micro‐, meso‐, and macro‐pores, implying the existence of a hierarchically porous structure of the fibers. It could be seen that the majority of pores were situated within the mesoporous range (Figure 4h). Besides, the maximum specific surface areas of the C‐SiC fibrous aerogels were measured as 373.23 m^2^ g^−1^ (Table S4).

Based on the preceding analysis, the mechanisms influencing the thermal conductivity of aerogels were comprehensively summarized (Figure 4i). The thermal conductivity is governed by the thermophysical properties of its constituent components as well as the geometric configuration of its aggregates [46]. Heat transfer occurs via four primary mechanisms: solid conduction, gas conduction, thermal convection, and thermal radiation. Firstly, the thermal conductivity of C‐SiC fibrous aerogels was predominantly controlled by solid conduction. Specifically, as the momordica charantia‐like C‐SiC fibers inside the aerogel architecture were arranged in a random orientation, heat was conducted through the ultrafine C‐SiC fibers network, extending the transmission path. Additionally, significant interfacial thermal resistance (Kapitza resistance) arose from the overlapping and entanglement of the fibers. Furthermore, the fibers comprised numerous C‐SiC interfaces and many defects, resulting in abundant phonon scattering sites [34]. The loose cross‐sectional characteristics of momordica charantia‐like fibers endowed the aerogels with an extremely low overall density and a minimal proportion of the solid skeleton, which further limited the number of effective carriers for solid‐phase heat conduction. These factors collectively contributed to the reduction of solid conduction. The momordica charantia‐like fibers possessed a hierarchically porous structure, with average pore sizes ranging from 4.6 to 7.1 nm. The Knudsen effect induced boundary scattering, which substantially diminished gas thermal conductivity [47]. Thirdly, the pore sizes were considerably smaller than the threshold for natural convection (approximately 1 mm), which could effectively suppress convective heat transfer [48]. Fourthly, the thermal radiation depended primarily on the material's optical properties and internal structure. Both SiC and C exhibited strong infrared shielding capabilities [49], characterized by high infrared absorption coefficients. Especially, in the C‐SiC fiber, free carbon predominantly existed in the sp^2^ hybridized form, establishing conductive pathways that increased free electron concentration. The interaction between free electron oscillations and incident electromagnetic waves enhanced absorption, resulting in a high infrared absorption coefficient to reduce thermal radiation. Consequently, when compared with other aerogels reported in the literature, the C‐SiC fibrous aerogel in this work demonstrated the lowest thermal conductivity (Figure 4j and Table S5), thereby underscoring the superior thermal insulation performance.

### 2.5 Electromagnetic Shielding Performance

**FIGURE 5:**
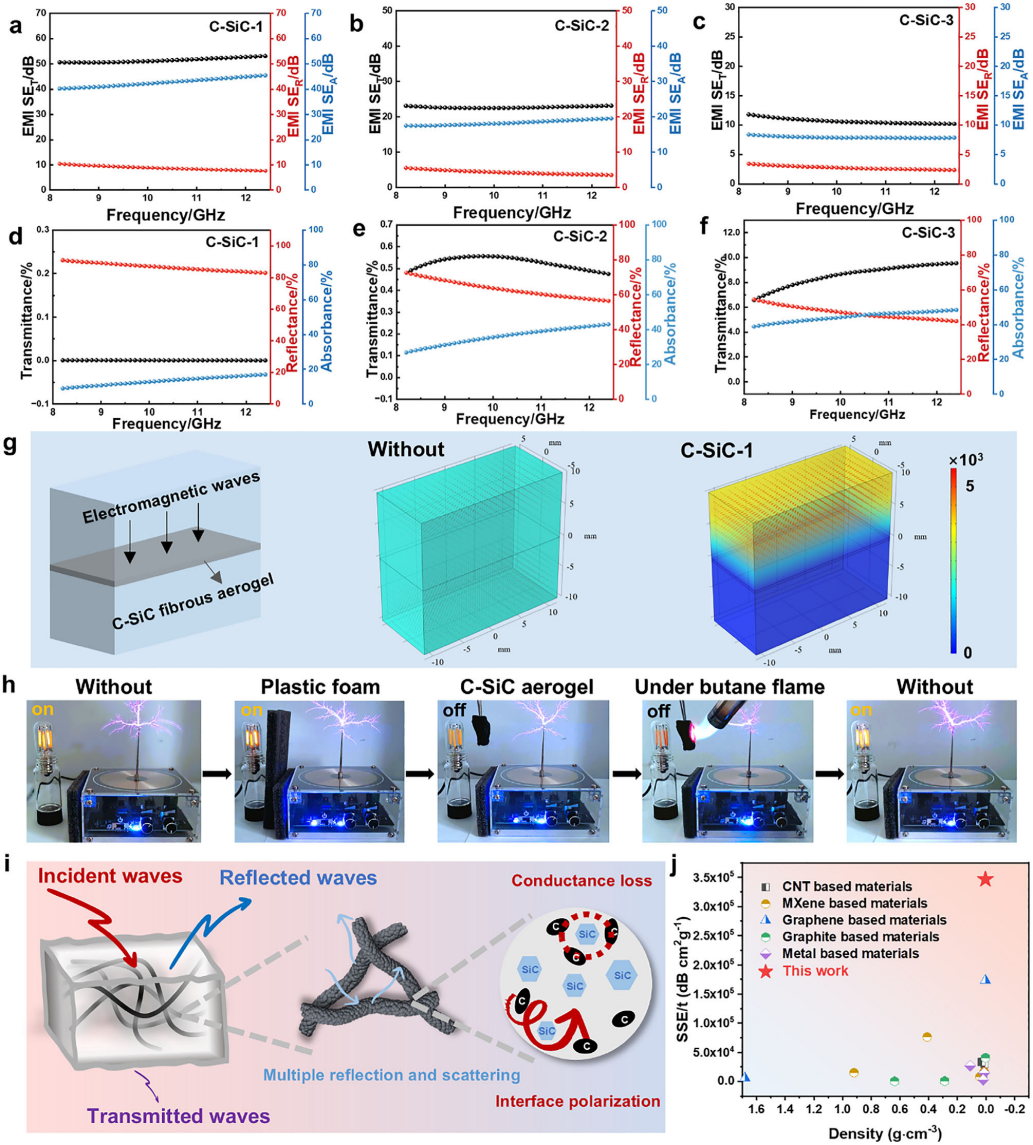
Electromagnetic shielding performance of C‐SiC fibrous aerogels. (a–f) The electromagnetic shielding performance, reflectivity, absorptivity, and transmittance ofC‐SiC‐X fibrous aerogels in the X‐band. (g) The simulation results of electric field distribution without and with C‐SiC fibrous aerogel. (h) The demonstration of EMI shielding performance based on a Tesla coil. (i) Schematic diagram of the electromagnetic shielding mechanism. (j) Comparison of the density and absolute electromagnetic shielding ability of this work with those reported in the literature.

The EMI shielding performance of the C‐SiC fibrous aerogels in the X‐band frequency range was systematically investigated (Figure 5a–c). It was observed that as the ratio of polysiloxane to PAN decreased, the carbon content of the C‐SiC fibrous aerogel correspondingly increased, leading to an improvement in the total electromagnetic shielding effectiveness (SE_T_). Notably, the C‐SiC‐1 fibrous aerogel exhibited the highest total SE_T_ of 53.1 dB. The specific shielding effectiveness normalized by thickness (SSE/t) was calculated based on the average SE_T_, incorporating both material thickness (t) and density (ρ), as defined by Equation (3) [20]. This parameter provides a normalized evaluation of the shielding efficiency of lightweight and thin materials. The SSE/t value for the C‐SiC‐1 fibrous aerogel, with a density of 3.9 mg cm^−3^ and a thickness of 0.38 mm, reached 346 828.6 dB cm^2^ g^−1^ (Table S6).

(3) $$\frac{SSE}{t}=\frac{SE_{T}}{\rho t}$$

The transmittance (T), reflectance (R), and absorptance (A) of the C‐SiC fibrous aerogels for electromagnetic waves of the X‐band were determined using Equations (4) and (5) [50] (Figure 5d,f). For the C‐SiC‐1 and C‐SiC‐2 samples, reflection contributed more strongly than absorption to the measured shielding behavior under the tested conditions. For the C‐SiC‐3 fibrous aerogel, reflection and absorption exhibited comparable contributions to the measured shielding response.

Specifically, in the frequency range of 8–10.4 GHz, reflectance slightly exceeded absorptance, suggesting a relatively stronger contribution from reflection than absorption. Conversely, in the frequency interval from 10.4 to 12.4 GHz, absorptance marginally surpassed reflectance, suggesting a relatively stronger contribution from reflection than absorption, suggesting an increased contribution from absorption under this frequency range. Furthermore, the reflectance of all C‐SiC fibrous aerogels exhibited a gradual decline with increasing frequency [51].

(4) $$SE_{T}=-10log_{10}\left(T\right)$$

(5) $$SE_{R}=-10log_{10}\left(1-R\right)$$

To gain more intuitive understanding of the EMI shielding capabilities of the fibrous aerogels, finite element simulations were conducted to assess the electric field distribution when electromagnetic energy was obstructed by the C‐SiC fibrous aerogels (Figure 5g). In the absence of any shielding material, electromagnetic waves transmitted freely without any attenuation of energy. Upon introduction of the C‐SiC‐1 fibrous aerogel, the electric field was nearly extinguished, demonstrating superior EMI shielding performance. Similarly, the C‐SiC‐2 and C‐SiC‐3 samples exhibited the capacity to impede and diminish the propagation of electromagnetic energy (Figure S39), findings that were corroborated by the aforementioned experimental data.

Additionally, the practical applicability of the C‐SiC fibrous aerogels was also tested (Figure 5h and Movie S2). A Tesla coil was employed, which could wirelessly illuminate a light bulb through the coupling effect of a high‐frequency, high‐voltage alternating electromagnetic field [52]. When a conventional plastic foam was positioned between the Tesla coil and the light bulb, the bulb continued to emit light, indicating the absence of electromagnetic shielding properties of the foam. In contrast, placement of the C‐SiC fibrous aerogel between the coil and the bulb resulted in the cessation of light emission, confirming the aerogel's effective electromagnetic shielding capability. Crucially, under the ablation of butane flame (∼1300°C), the C‐SiC fibrous aerogel not only insulated the light bulb from the rapidly rising ambient temperature, but also exhibited electromagnetic shielding performance, rendering the light bulb unlit. When the butane flame and C‐SiC fibrous aerogel were subsequently removed, the light bulb remained undamaged and continued to illuminate, confirming the excellent dual functions of thermal insulation and electromagnetic shielding performance of the fibrous aerogel. In addition, the C‐SiC fibrous aerogel maintained its electromagnetic shielding performance after being corroded in 1 M HCl and 7 M NaOH for 7 days, with SSE/t values of 48 084.4 dB cm^2^ g^−1^ and 64 283.8 dB cm^2^ g^−1^, respectively (Figures S40 and S41). The superior EMI shielding performance of the C‐SiC fibrous aerogels could be ascribed to the synergistic interplay between their structural features and compositional attributes (Figure 5i and Table S7). The aerogel possessed a 3D architecture characterized by randomly interwoven momordica charantia‐like fibers. The surface exhibited a complex morphology of pores of varying sizes, which facilitated multiple internal reflections and scatterings of electromagnetic waves within the material [53]. This phenomenon not only prolonged the propagation path of the waves but also enhanced the likelihood of energy dissipation. Concurrently, the carbon component of the aerogels established a continuous conductive network. Upon incidence of electromagnetic waves, the impedance mismatch between air and this conductive network induced reflection at the material's surface [54]. Moreover, the high electrical conductivity amplified interfacial charge accumulation, thereby further augmenting reflection efficiency [55]. Additionally, the heterogeneous interface formed between SiC and C engendered interfacial polarization, which intensified the local electric field and promoted energy dissipation [56]. Besides, SiC could convert electromagnetic wave energy into thermal energy through mechanisms such as dipole polarization and conduction loss. The carbon conductive network also contributed to electromagnetic energy attenuation via the eddy‐current effect. Most importantly, the momordica charantia‐like fibers enhanced electromagnetic wave dissipation via their structure with a dense exterior and a loose interior, which also reduced the weight of the aerogel, thus achieving an excellent performance profile featuring ultralow density combined with ultrahigh specific electromagnetic shielding effectiveness. Compared with previously reported studies, this work represents a significant advancement by achieving materials with the highest SSE/t (Figure 5j and Table S8). Thereof, the C‐SiC fibrous aerogels break the trade‐off between thermal insulation and electromagnetic shielding performance, demonstrating enormous application prospects in high‐tech fields for extreme environments.

## 3 Conclusions

In summary, instead of resorting to expensive fillers and sophisticated fabrication protocols, this work proposed a facile and cost‐effective strategy for directly constructing integrated‐architecture materials using low‐cost polysiloxane and PAN as dual precursors. By virtue of preregulated structural design paradigm, 3D polymer fibrous aerogels were successfully prepared via centrifugal spinning technology. Organic moieties, oxygen, and nitrogen species were gradually removed through subsequent thermal pyrolysis and sintering, ultimately yielding the flexible C‐SiC fibrous aerogels. Benefiting from the successful implementation of the biomimetic strategy, the momordica charantia‐like fibers, featuring a loose internal structure and relatively dense exterior, innovatively achieve weight reduction and performance enhancement. The aerogels with ultralow density of 3.9 mg cm^−3^ exhibited an ultralow thermal conductivity of 9.12 mW m^−1^ K^−1^, which also attained a maximum electromagnetic SE of 53.1 dB and an ultrahigh SSE/t of 346 828.6 dB cm^2^ g^−1^. Moreover, the aerogels possessed a suite of comprehensive properties, including compressible resilience, acid‐alkali corrosion resistance, ultrahigh‐temperature stability (2000°C), and oxidation resistance (1000°C). Collectively, this work establishes a scalable and economical route for the fabrication of multifunctional flexible C‐SiC fibrous aerogels, which not only breaks the trade‐off between thermal insulation and electromagnetic shielding performance, but also shows great promise for advancing the development of integrated functional materials, especially for potential aerospace applications.

## 4 Experimental Section

### 4.1 Materials

Polysiloxane and Polyacrylonitrile (PAN) were obtained from our lab. N, N‐Dimethylacetamide (DMAc) was from Sinopharm Chemical Reagent Co., Ltd. Polyvinylpyrrolidone (PVP) was purchased from Shanghai Aladdin Biochemical Technology Co., Ltd.

### 4.2 Fabrication of the C‐SiC Fibrous Aerogel

Three groups of spinning solution with different mixing ratios of polysiloxane to PAN (0.5, 2.0, and 3.0, respectively) were prepared. The spinning solution was stirred at 60°C for 5 h before usage. The thoroughly mixed spinning solution was transferred to the spinneret of the centrifugal spinning apparatus. The operational parameters were configured as follows: an applied voltage of −30 kV, a collection distance ranging from 20 to 25 cm, and a liquid feed rate between 30 and 40 mL/h. A needle gauge of 24 G or larger was selected. The rotating cup speed was maintained between 1600 and 2000 rpm, with the temperature controlled at 23°C and relative humidity adjusted to 60%. Subsequently, the resulting polymer fibrous aerogel was dried in an oven at 60°C for 5 h to eliminate residual solvent, yielding the polymer fibrous aerogel. Then, the polymer fibrous aerogel was irradiated on both sides using an ultraviolet (UV)‐LED lamp for durations ranging from 2 to 12 h. The preliminarily treated polymer fibrous aerogel was subjected to air curing in a pre‐oxidation furnace for 2 h to produce the fully cured fibrous aerogel. The cured fibrous aerogel was positioned in a graphite furnace and subjected to pyrolysis treatment by heating it to 1300°C under an inert atmosphere, resulting in the formation of Si‐C‐O‐N fibrous aerogel. Subsequently, the Si‐C‐O‐N fibrous aerogel underwent high‐temperature sintering at 1800°C in an inert atmosphere to produce the C‐SiC fibrous aerogels (named as C‐SiC‐1, C‐SiC‐2 and C‐SiC‐3 according to the different mixing ratio of polysiloxane to PAN, respectively).

### 4.3 Characterization

The oxygen and carbon content were measured by oxygen‐nitrogen analyzer (EMIA‐820, Horiba, Japan) and a carbon‐sulfur analyzer (EMIA‐320, Horiba, Japan), respectively. The measurement was repeated three times, and the average value obtained was taken as the oxygen/carbon content of the sample. The silicon content was quantitatively analyzed by colorimetry after dissolving the sample in molten strong alkali. The sample was ground into powder, and the sample amount was 0.5–1 g. The morphological analysis was conducted using scanning electron microscopy (SEM, TESCAN MIRA3, Czech Republic) and transmission electron microscopy (TEM, Titan G2 60–300, FEI, USA). Elemental mapping was determined via energy‐dispersive spectroscopy (EDS) integrated with the SEM. Fourier transform infrared spectroscopy (FTIR) was carried out using an infrared spectrometer (Frontier, PerkinElmer, USA). X‐ray diffraction (XRD) patterns were recorded on a Bruker AXS D8 Advance diffractometer (Bruker, Germany) equipped with Cu Kα radiation (λ = 1.54178 Å), with 2θ ranging from 10° to 80° at a scanning rate of 10°min^−1^. X‐ray photoelectron spectroscopy (XPS) analysis was performed using an Escalab 250Xi spectrometer (Thermo Fisher, USA) with an Al Kα excitation source (1487.6 eV). Raman spectra were obtained using a laser micro‐Raman spectrometer (Renishaw inVia, Renishaw, UK) with a 532 nm laser. Mechanical compression tests were conducted using a universal testing machine (MTS, CMT4503, USA) equipped with a 20 N load cell. Solid‐State Nuclear Magnetic Resonance (SS‐NMR) was conducted on an NMR spectrometer (AVANCE NEO 400 MHz, Bruker, Germany). Specific Surface Area and Pore Size Analyzer was used to analyze the adsorption amount of N_2_ gas under different relative pressures (BSD‐PM1/2, Germany). The BET theory and the NLDFT (Non‐Local Density Functional Theory) model were employed to calculate the specific surface area, pore volume, and pore size distribution. The X‐ray computed tomography (X‐CT) was used to analyze 3D morphology and porosity (EasyTom 160 Micro, France).

The thermal conductivity was measured using the laser flash method on a laser flash apparatus (LFA 467HT HyperFlash, Netzsch, Germany). For the analysis, the C‐SiC fibrous aerogel was cut into square specimens with size of 9.8 mm × 9.8 mm.

(6) $$\lambda =\alpha \;\times \;C_{p}\;\times \;\rho$$

Where *λ* is the thermal conductivity (W/m·K), *ρ* is the density (kg/m^3^) obtained by the geometric method, *C*_p_ is the specific heat capacity (J/kg·K) measured by Differential scanning calorimetry.

Vector Network Analyzer (VNA) method was used to measure the electromagnetic shielding effectiveness (SE). This method is based on the transmission line theory, and the electromagnetic shielding effectiveness of materials is calculated by measuring the reflection (S_11_) and transmission (S_21_) parameters of materials for electromagnetic waves. The calculation formula for SE is as follows:

(7) $${\mathrm{SE}}_{R}=-10\log_{10}\left(1-{\left|S_{11}\right|}^{2}\right)$$

(8) $${\mathrm{SE}}_{A}=-10\log_{10}\left(\frac{{\left|S_{21}\right|}^{2}}{1-{\left|S_{11}\right|}^{2}}\right)$$

(9) $${\text{SE}}_{T}=-10\;\mathrm{lo}g_{10}\left({\left|S_{21}\right|}^{2}\right)$$

The vector network analyzer was used for testing (E5071C, Agilent, USA). The material to be tested was processed into square sheets of specific sizes with a thickness of 0.3–3 mm. The surface of the sample was ensured to be flat to allow the electromagnetic waves to be incident uniformly.

## Author Contributions

Conceptualization: Y.G., T.Y. and Y.S. Methodology: T.Y., Y.S., Z.Z., Z.X. and Y.G. Investigation: T.Y., Y.S., Q.C. and X.W. Visualization: T.Y., Y.S., C.Z., Z.L. and Y.Z. Supervision: Y.G. Writing the original draft: T.Y. and Y.S. Writing, review & editing: Y.G. Funding acquisition: Y.G.

## Conflicts of Interest

The authors declare no conflicts of interest.

## Supporting information




**Supporting File 1**: advs76343‐sup‐0001‐SuppMat.docx.

**Supporting File 2**: advs76343‐sup‐0002‐MovieS1.mp4.

**Supporting File 3**: advs76343‐sup‐0003‐MovieS2.mp4.

## Data Availability

The data that support the findings of this study are available in the supplementary material of this article.
