# Supplementary material for: Bioinspired Multifunctional Flexible C‐SiC Fibrous Aerogel for Superior Electromagnetic Interference Shielding Under Extreme Environments
Source: Adv Sci (Weinh). 2026 Jul 7:e76343. Online ahead of print. doi: 10.1002/advs.76343 (PMC13339101; doi:10.1002/advs.76343)
Supplement: Supplementary file 1 — Supporting File 1: advs76343‐sup‐0001‐SuppMat.docx. [file ADVS-9999-e76343-s002.docx]

Supporting Information

Bioinspired Multifunctional Flexible C-SiC Fibrous Aerogel for Superior Electromagnetic Interference Shielding Under Extreme Environments

Tianyue Yang, Yan Shen, Zhongqian Zhao, Xue Zhou, Qianji Chen, Xujing Wang,

Chuchu Zheng, Zhaoxiang Lu, Yangzhong Zhao, and Yanzi Gou*

The inner diameter of the spinneret (needle) needs to be selected according to the rheological properties of the spinning solution. Generally, a spinning solution with lower viscosity is suitable for a spinning needle with a finer inner diameter. The spinning solution of the polysiloxane/PAN system was not suitable to form fibers in a 22G needle (*ϕ*_inner_ = 0.41 mm), which could form fibers in 24-26G needles (*ϕ*_inner_ = 0.24-0.31 mm).

**
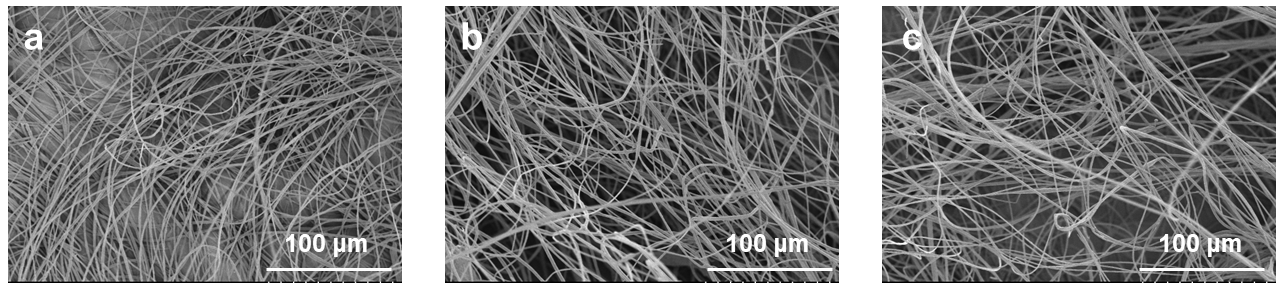
**

**Figure S1.** SEM images of polymer fibrous aerogel obtained with different needle diameters: (a) 24G, (b) 25G, (c) 26G.

Temperature and humidity show a significant impact on the three-dimensional structure of fibrous aerogels during the centrifugal spinning process. At 23 °C with 60 RH%, the polymer fibrous aerogel exhibited a fluffy three-dimensional structure, with fibers entangled and interlaced. The distance between the fibers was relatively large, with a spacing of up to several tens of micrometers. The fibrous aerogel exhibited a low solid content, low density, and high morphological quality. When the humidity was increased to 75 RH% at a constant temperature, the fibrous aerogel still maintained a three-dimensional structure, but the fibers were moist and sticky. Especially under extremely high humidity conditions, although the microscopic structure of the fibrous aerogel remained unchanged, the fiber surface was rough and grooved, which was caused by the evaporation of residual solvents and condensed water on the fiber surface. However, when the humidity was reduced to 55 RH% or below, the three-dimensional structure of the fibrous aerogel disappeared. It appeared macroscopically as a two-dimensional membrane. And the in-plane deposition of the fibers was very dense. Therefore, 23 °C and 60 RH% were the optimal temperature and humidity condition for the centrifugal spinning of the polysiloxane/PAN system.


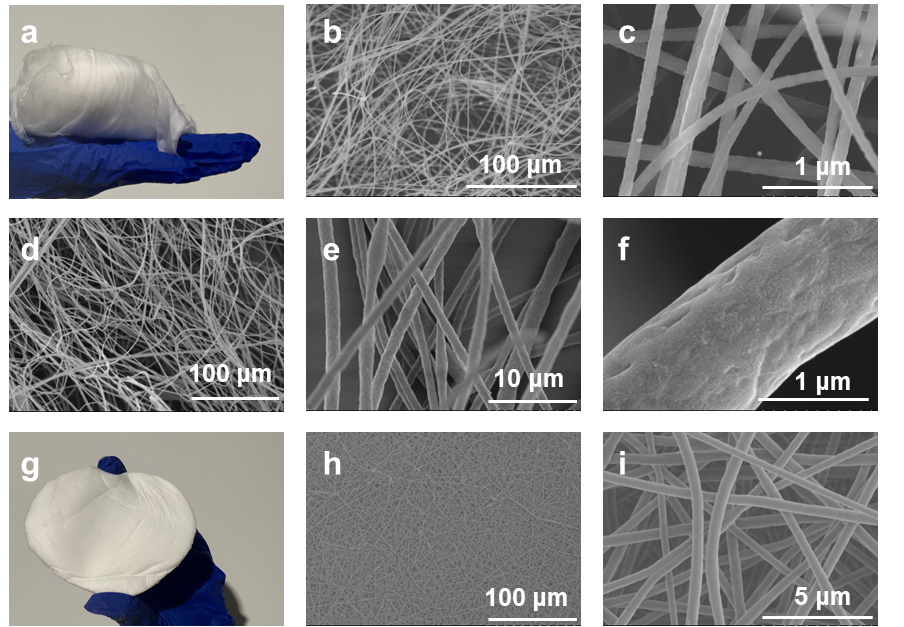


**Figure S****2.** Optical images (a, g) and SEM images of polymer fibrous aerogel obtained at 23 ^o^C with different humidity: (a-c) 60 RH%; (d-f)75 RH%; (g-i) 55 RH%.

The shape and gap of the collection device also influence the three-dimensional structure of the fibrous aerogel. During the spinning process of the Sample-1 solution, the fiber deposition characteristics were similar to those of pure PAN fibers. The fibers were long and thin, which deposited densely. Moreover, even with a collection distance close to 30 cm, it was still difficult to overcome the problems of hanging and entanglement of the long and thin fibers. Therefore, a vertical wind field had to be used to assist the deposition. The shape of the original collector was shown in Figure S3(a, b), and the improved device was shown in Figure S3(c, d). On the basis of not changing the planar collection, changing the shape and increasing the gap could also effectively improve the fiber collection situation.

**
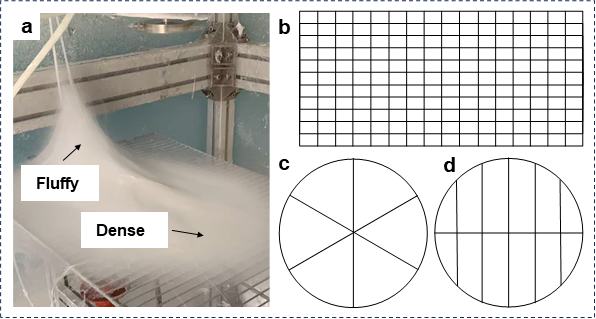
**

**Figure S3.** (a) Optical image of polymer fibrous aerogel and (b-d) Schematic diagrams of planar collection devices with gaps of different shapes.

**
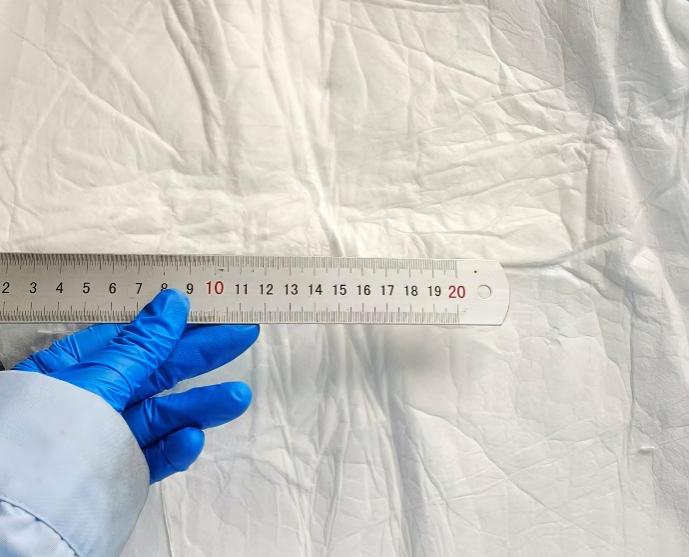
**

**Figure S4.** Optical photograph of polymer fibrous aerogel.

By regulating the spinning conditions, high-quality polymer fibrous aerogels could be obtained from spinning solution with three different polysiloxane/PAN ratios. The fibers show relatively uniform diameter (~1 μm), smooth surface without obvious beads.

**
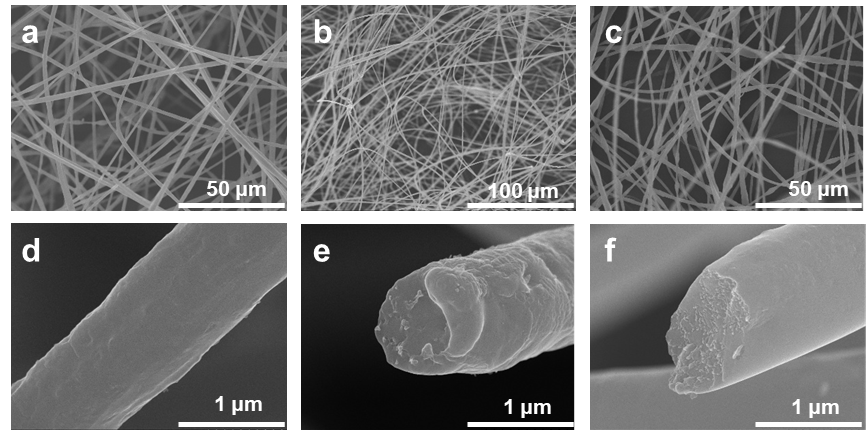
**

**Figure S5.** SEM images of polymer fibrous aerogels (named as PFA): (a) PFA-1, (b) PFA-2, (c) PFA-3, (d-f) Fiber surface and cross-section of PFA-1 fibrous aerogel under high magnification.

To further clarify the curing mechanism, TG analysis was conducted on polysiloxane before and after UV irradiation. The original polysiloxane showed a high ceramic yield. The ceramic yield at 1300 °C was 81.3%. However, the softening point of polysiloxane was low, which was even lower than the thermal cross-linking temperature. Therefore, during the pyrolysis process, polysiloxane fiber was prone to melting, leading to fiber coalescence, which was not conducive to maintaining the fiber structure. UV irradiation could open the benzene rings of polysiloxane and complete the self-crosslinking process to avoid occurrence of melting before pyrolysis. Meanwhile, the ceramic yield of the cured polysiloxane at 1500 °C increased from 75% to 83%.

**Figure S6.** Ceramic yield of polysiloxane before and after UV irradiation.

After UV curing, the ceramic yield of polysiloxane at 1300 °C was as high as 83%. The temperature range of main weight loss was between 500-650 °C, and the maximum exothermic peak was located at 840 °C. After air curing, the mass retention of PAN at 1300 °C was about 45%, and the maximum exothermic peak was located between 1000-1200 °C.


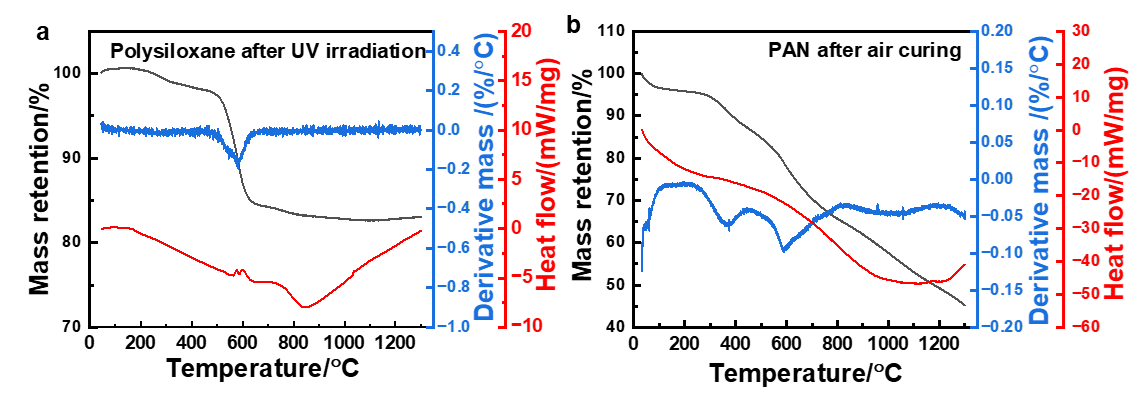


**Figure S7.** (a) TG-DTG-DSC curves of UV-irradiated infusible polysiloxane, (b) TG-DTG-DSC curves of PAN cured in air at 260 °C.


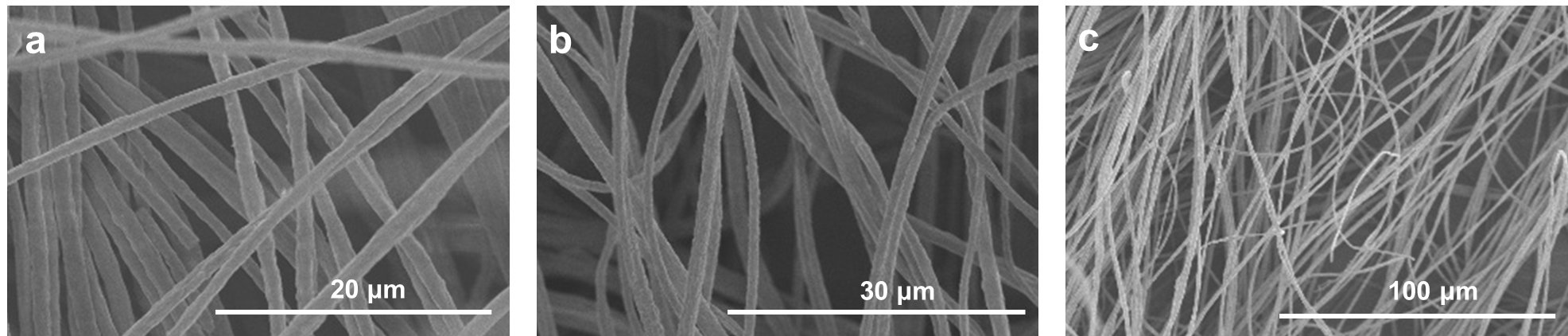


**Figure S8.** SEM images of cured fibrous aerogels (named as CFA): (a) CFA-1, (b) CFA-2, (c) CFA-3.

FT-IR spectra of polysiloxane, PAN, polymer fibrous aerogel, cured fibrous aerogel, and Si-C-O-N fibrous aerogel is shown in Figure S9. The peak at 3072 cm⁻¹ was the C-H stretching vibration peak of the phenyl group. The peak at 1430 cm⁻¹ was the C=C stretching vibration of the phenyl group, and the peak at 1041 cm⁻¹ was the stretching vibration of Si-Ph. The peak at 1007 cm⁻¹ was the vibration peak of Si-O-Si, which could be attributed to the main chain of polysiloxane. The peak at 2244 cm⁻¹ was the stretching vibration of -CN. After the curing treatment, the-CN peaks almost completely disappeared. The vibration at 1598 cm⁻¹ was the vibration of cyclic conjugated -C=N-, which was generated in large quantities during the cyclization and cross-linking process of PAN. In addition, the vibration peaks of the benzene ring were all weakened to a certain extent. After pyrolysis, the vibration peaks of organic groups represented by the C-H stretching vibration at 3045 cm⁻¹ and the C-H in-plane deformation at 1276 cm⁻¹ all disappeared.

**Figure S9.** FT-IR spectra of polysiloxane, PAN, polymer fibrous aerogel, cured fibrous aerogel, and Si-C-O-N fibrous aerogel.

**
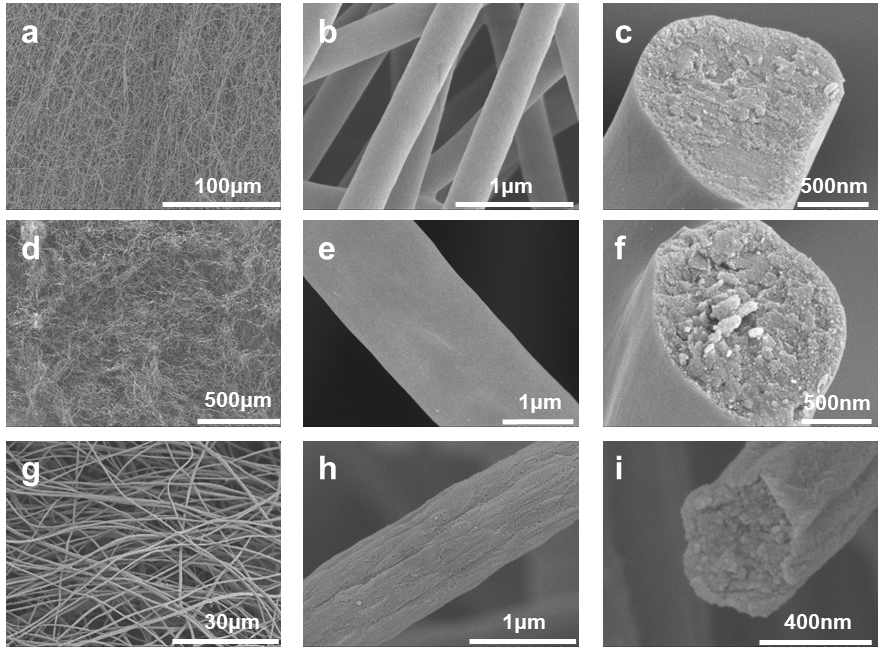
**

**Figure S10.** SEM images of inorganic Si-C-O-N fibrous aerogels: (a-c) Si-C-O-N-1, (d-f) Si-C-O-N-2, (g-i) Si-C-O-N-3.


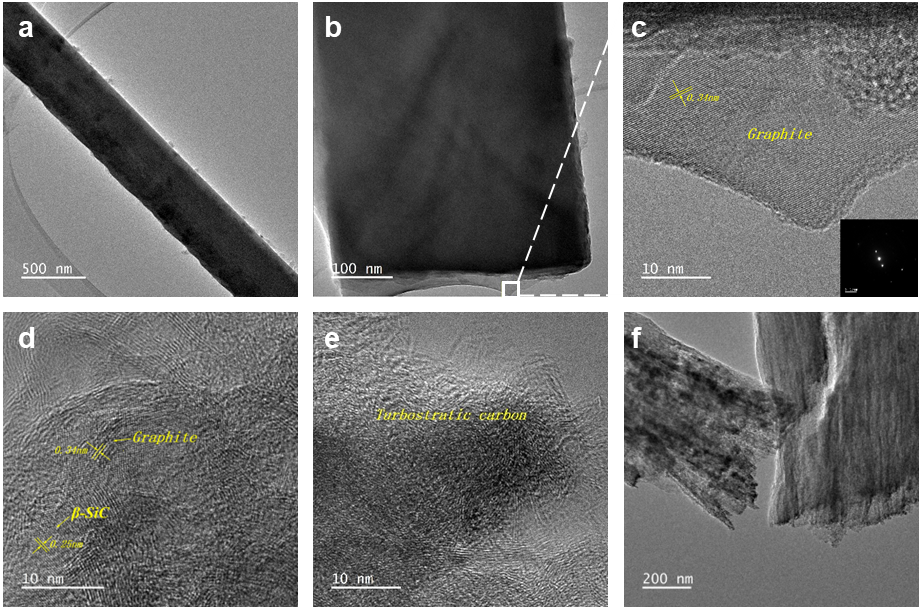


**Figure S11.** TEM images of the Si-C-O-N fibrous aerogel: (a, f) Fiber morphologies at low magnification, (b, c) Microstructures of fiber fractures, and (d, e) HRTEM images of the Si-C-O-N fiber.

**
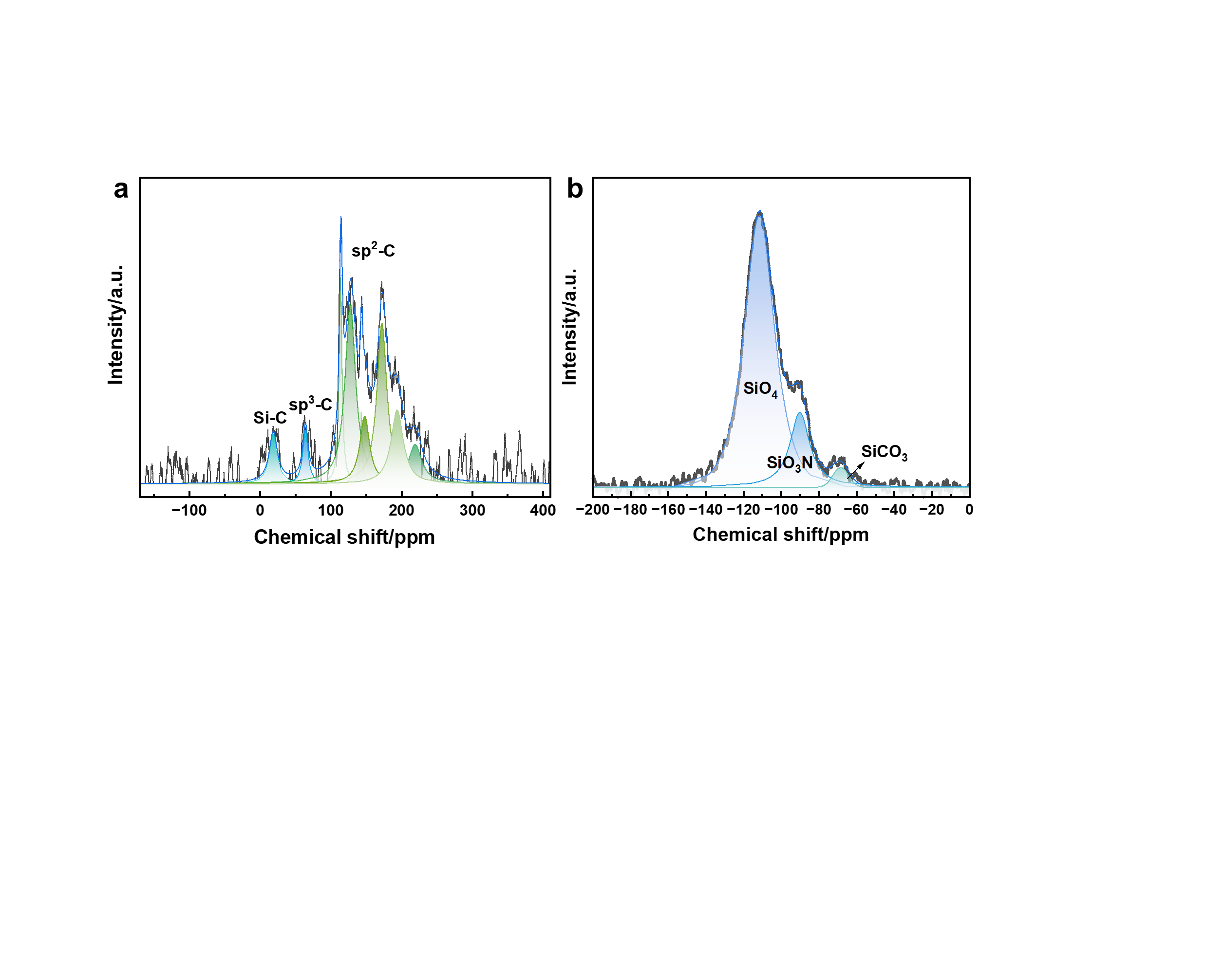
**

**Figure S12.** MAS NMR spectra of the Si-C-O-N fibrous aerogel: (a) ^13^C-NMR, (b) ^29^Si-NMR.

**
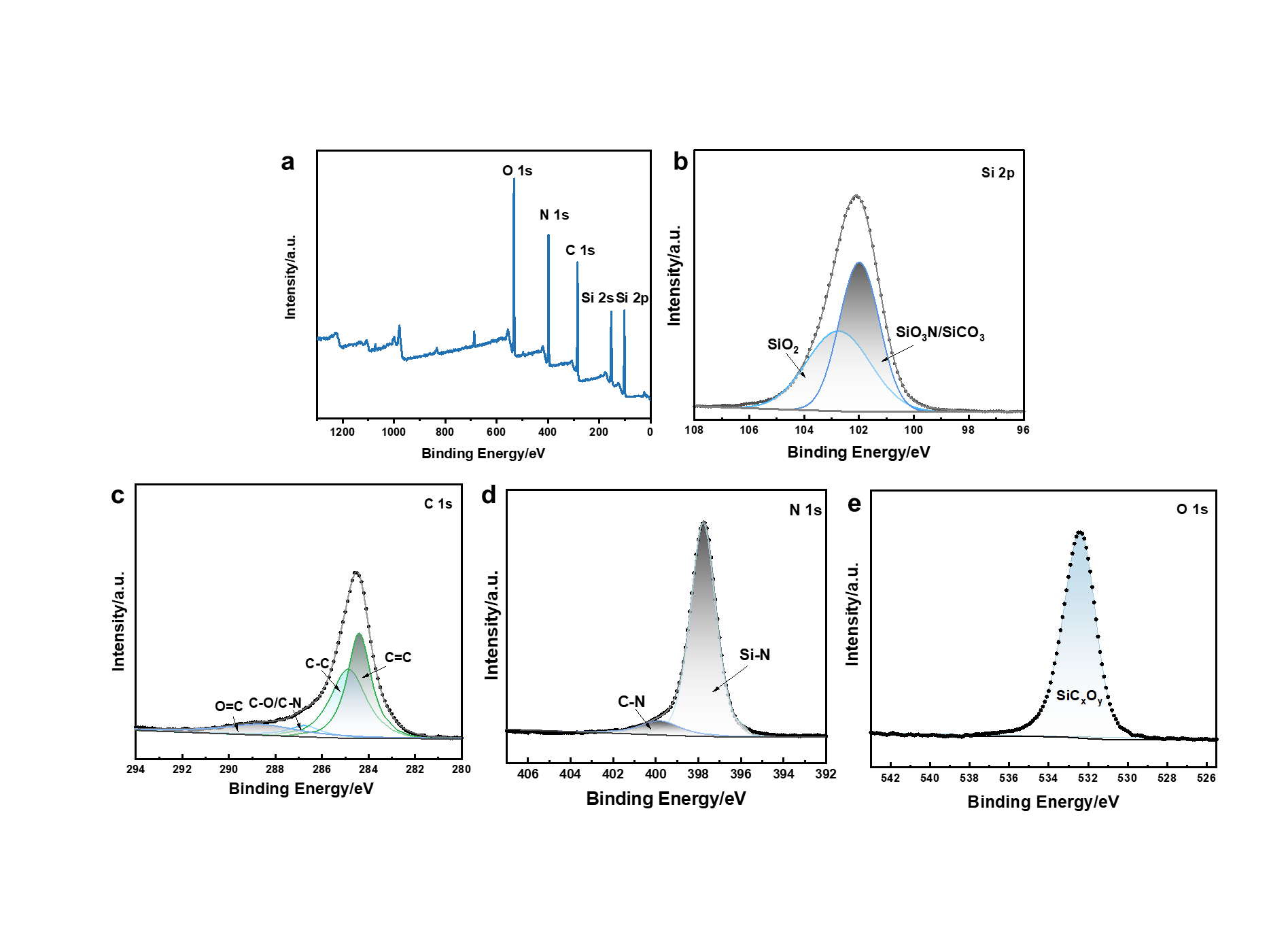
**

**Figure S13.** XPS spectra of the Si-C-O-N fibrous aerogel.

**
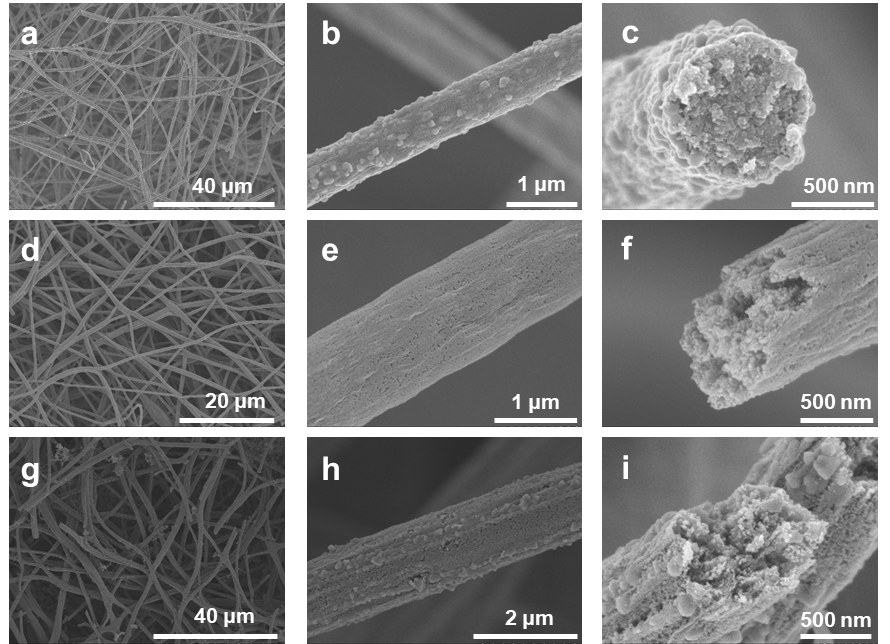
**

**Figure S14.** SEM images of fibrous aerogels prepared at 1600 °C (named as FA1600): (a-c) FA1600-1, (d-f) FA1600-2, (g-i) FA1600-3.


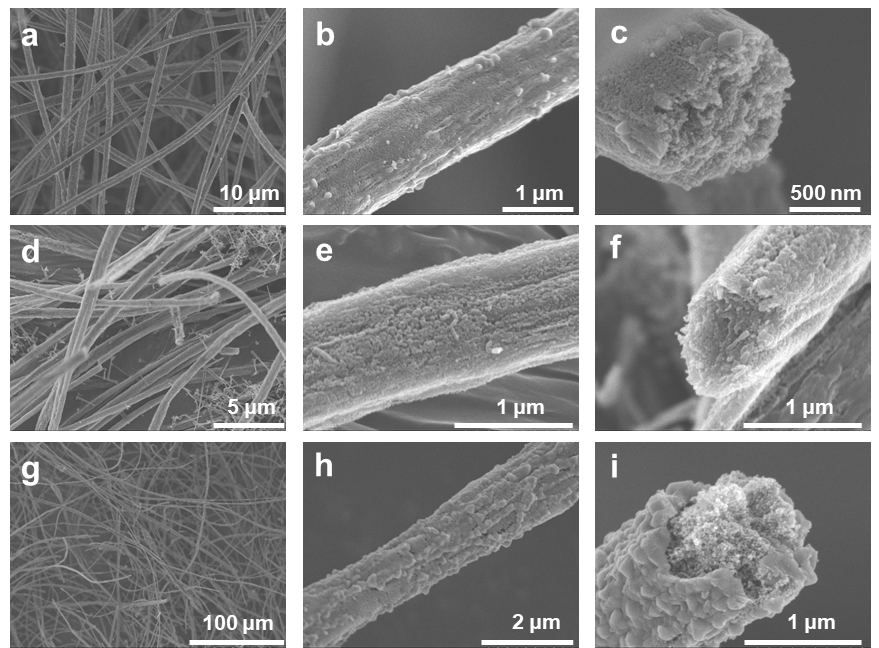


**Figure S15.** SEM images of the C-SiC fibrous aerogel prepared at 1800 °C: (a-c) C-SiC-1, (d-f) C-SiC-2, (g-i) C-SiC-3.


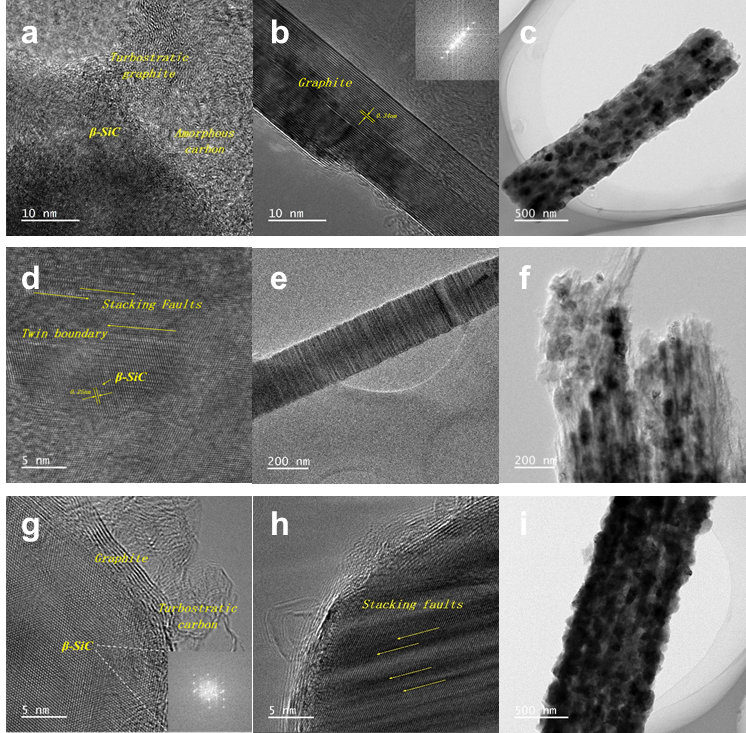


**Figure S16.** HRTEM and TEM images of fibrous aerogels obtained at 1600 ^o^C: (a-c) FA1600-1, (d-f) FA1600-2, and (g-i) FA1600-3.


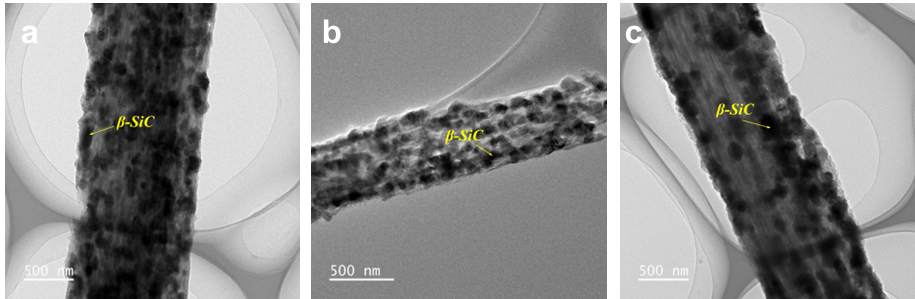


**Figure S17.** TEM images of the C-SiC fibrous aerogels: (a) C-SiC-1, (b) C-SiC-2, (c) C-SiC-3.


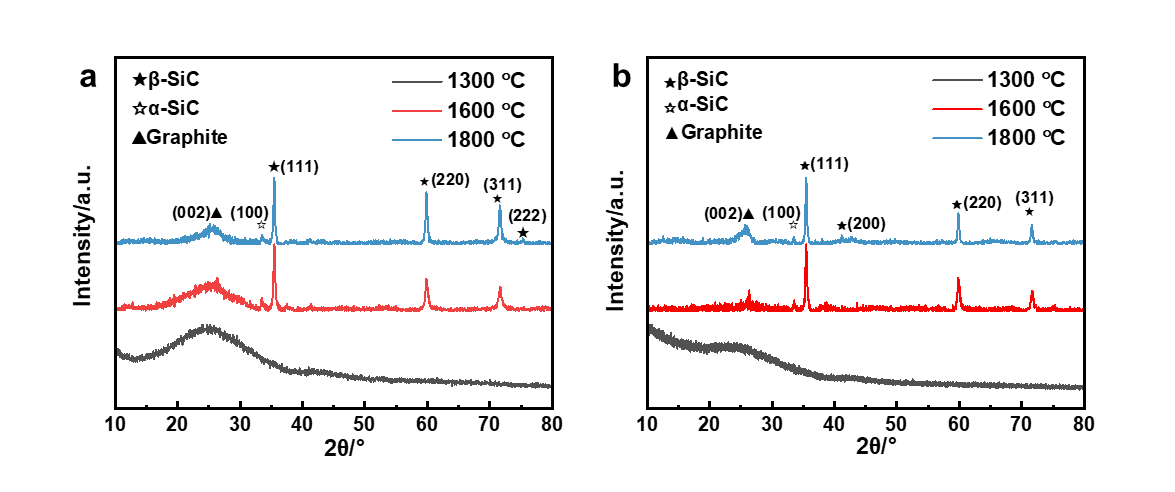


**Figure S18.** XRD patterns at each stage of the preparation of fibrous aerogels: (a) Si-C-O-N-1, FA1600-1 and C-SiC-1, (b) Si-C-O-N-2, FA1600-2 and C-SiC-2.

The C-SiC fibrous aerogels were further analyzed by Raman spectroscopy (Figure S19). The I_D_/I_G_ ratio (area ratio) was obtained by fitting the Raman spectrum. For the C-SiC fibrous aerogels prepared at 1800 °C, the I_D_/I_G_ decreased from 1.37 to 1.12 with the increment of polysiloxane/PAN. Meanwhile, the peak shape of the 2D peak became sharper, indicating that the proportion of defects in the free carbon decreased. As shown in Figure S20, the I_D_/I_G_ decreased with the increase of the preparation temperature, which reflected that increasing the temperature was beneficial to the ordering of free carbon. In addition, the SiC peaks of the fibrous aerogels could be seen at 793 cm⁻¹ and 970 cm⁻¹.

**Figure S19.** Raman spectra of the C-SiC fibrous aerogels.

**Figure S20.** Raman spectra of the fibrous aerogels obtained at different temperatures.

**Figure S21.** The tensile strength–tensile strain curve of C-SiC fibrous aerogel.

**
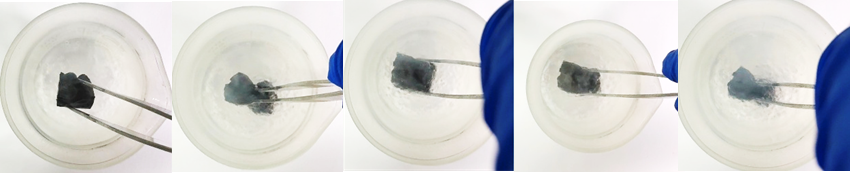
**

**Figure S22.** Optical images of compression performance display of the C-SiC fibrous aerogel in liquid nitrogen.

**
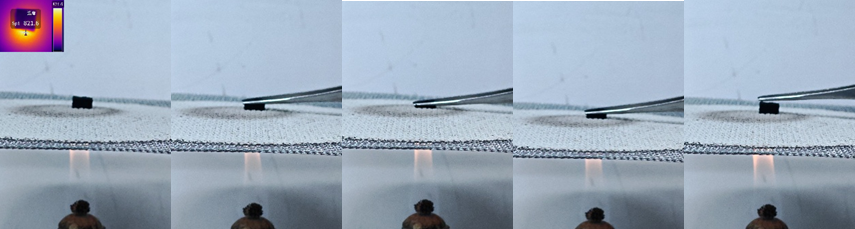
**

**Figure S23.** Optical images of compression performance display of the C-SiC fibrous aerogel being heated by an alcohol lamp.

**
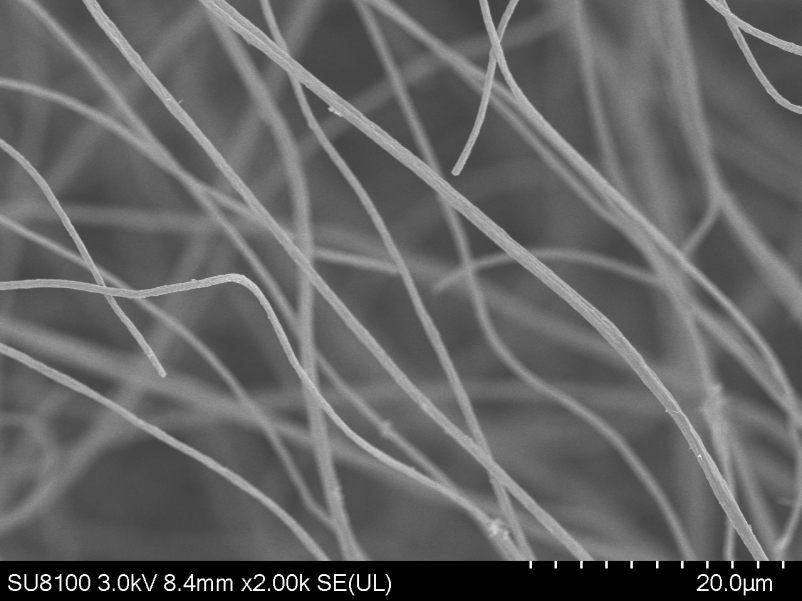
**

**Figure S24.** SEM image of the C-SiC fibrous aerogel after being held in 1 M HCl for 7 days.

**
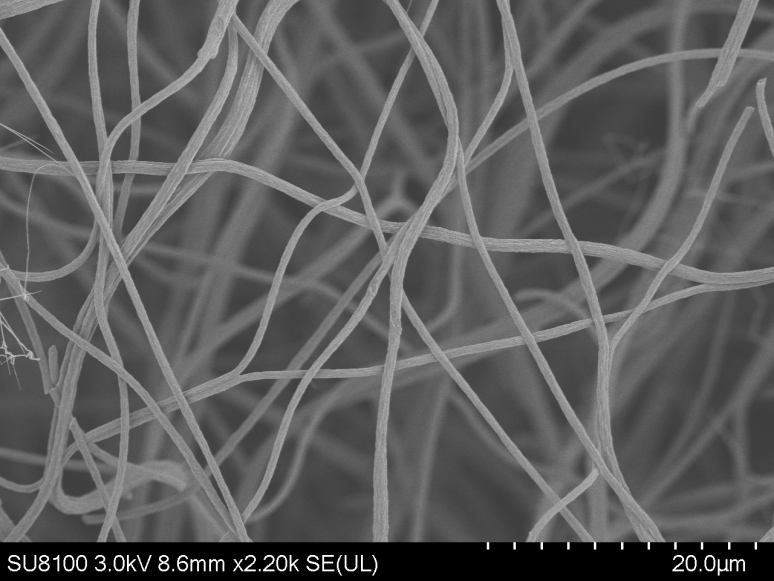
**

**Figure S25.** SEM image of the C-SiC fibrous aerogel after being held in 6 M NaOH for 7 days.


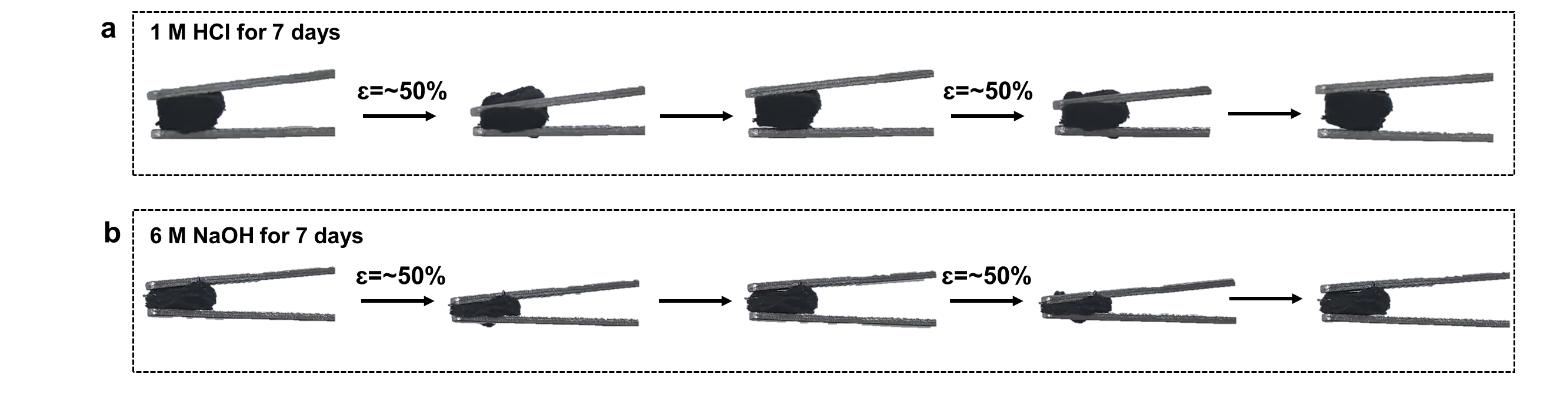


Figure S26. Demonstration of the mechanical performance of C-SiC fibrous aerogels after being held in (a) 1 M HCl for 7 days and (b) 6 M NaOH for 7 days.


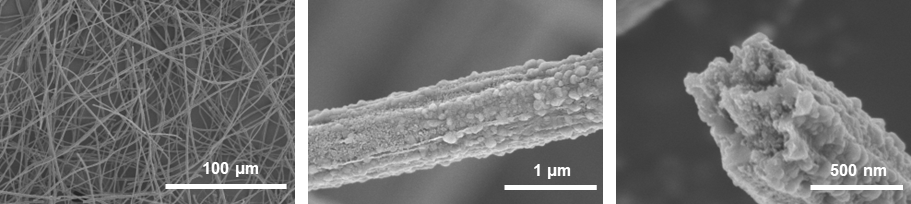


**Figure S27.** SEM images of the C-SiC fibrous aerogel after heat treatment at 1800 °C for 1 h in Ar atmosphere.


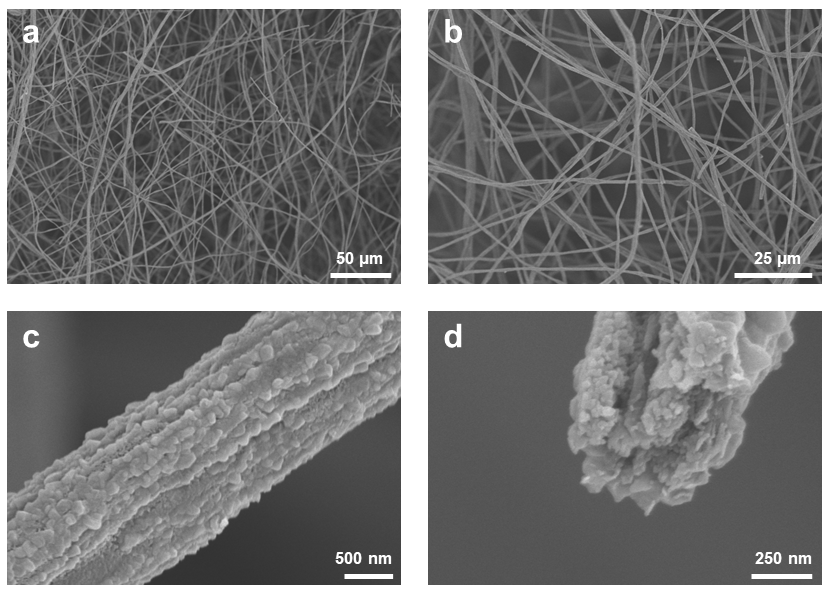


**Figure S28.** SEM images of the C-SiC fibrous aerogel after heat treatment at 1800 °C for 5 h in Ar atmosphere.

**Figure S29.** XRD pattern of the C-SiC fibrous aerogel after heat treatment at 1800 °C for 5 h in Ar atmosphere.


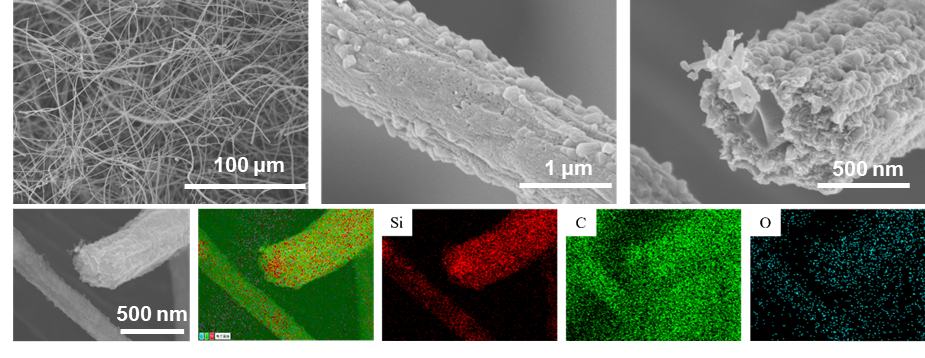


**Figure S30.** SEM images of the C-SiC fibrous aerogel after heat treatment at 2000 °C for 1 h in Ar atmosphere.

**
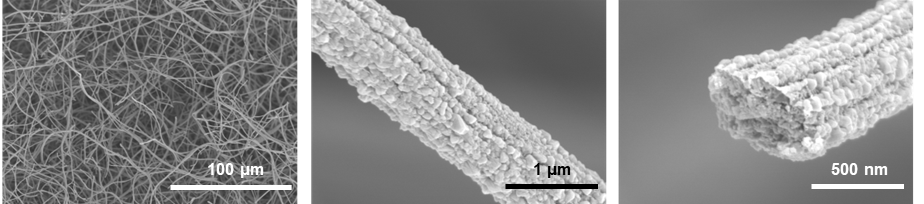
**

**Figure S31.** SEM images of the C-SiC fibrous aerogels after heat treatment at 2000 °C for 1 h in vacuum.

As shown in Figure S33, after oxidation at 700 ℃ for 1 h in air, the C-SiC fibrous aerogel remained intact with the color changing from black to white. However, the carbon fibrous aerogel almost disappeared with only tiny residue left. The comparison showed obvious improvement in oxidation resistance of the C-SiC fibrous aerogel.

**
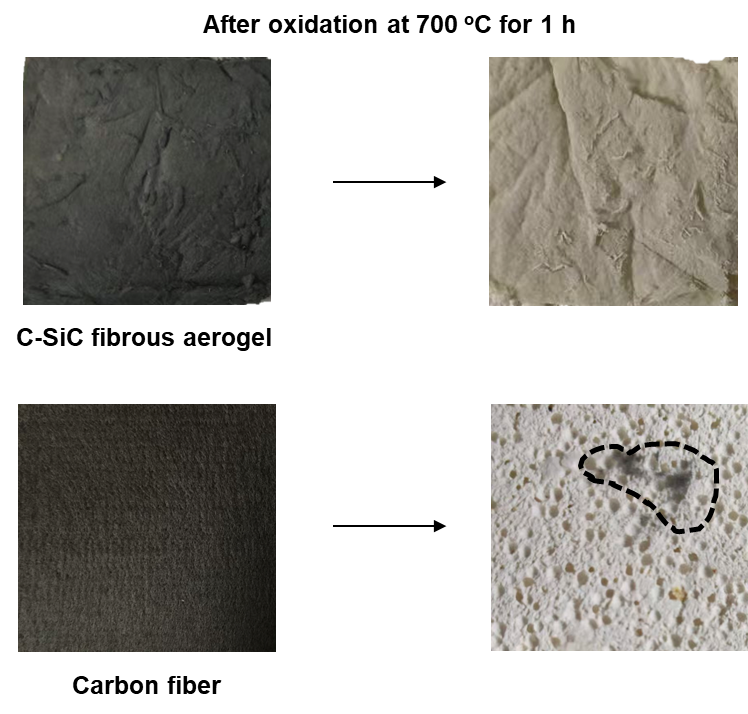
**

**Figure S32.** Photographs of the C-SiC fibrous aerogels after oxidation at 700 ℃ for 1 h in air.

**
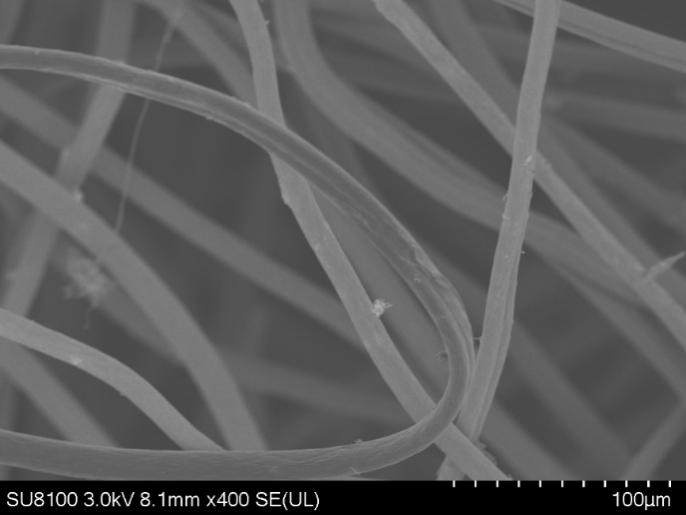
**

**Figure S33.** SEM image of carbon fibrous aerogel.

**
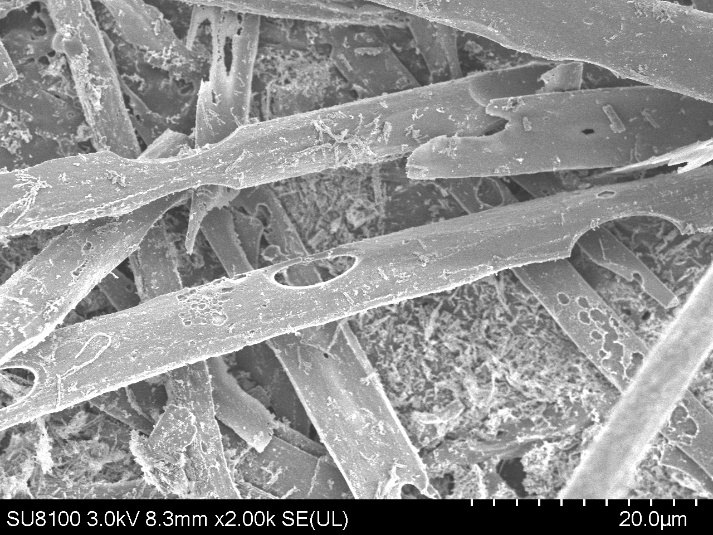
**

**Figure S34.** SEM image of the carbon fibrous aerogel residue after oxidation at 700 ℃ for 1 h in air.

**
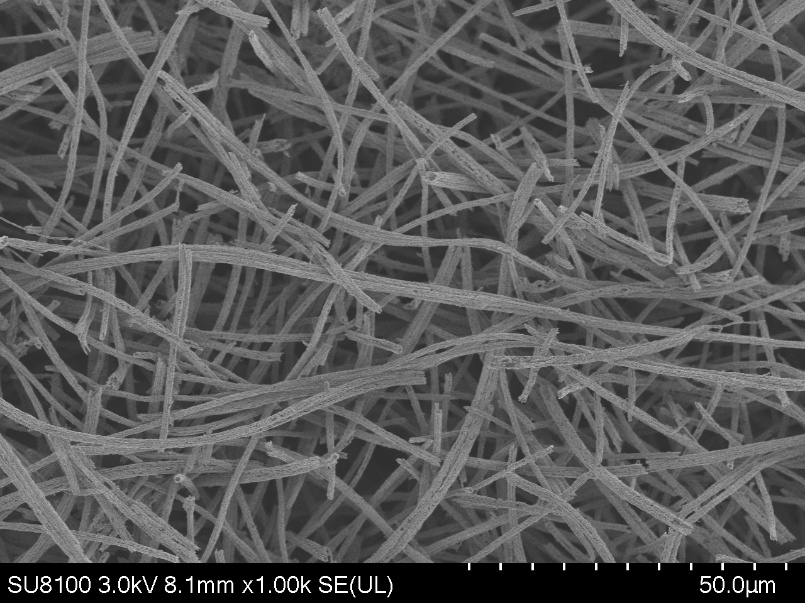
**

**Figure S35.** SEM image of the C-SiC fibrous aerogel after oxidation at 700 ℃ for 1 h.

**
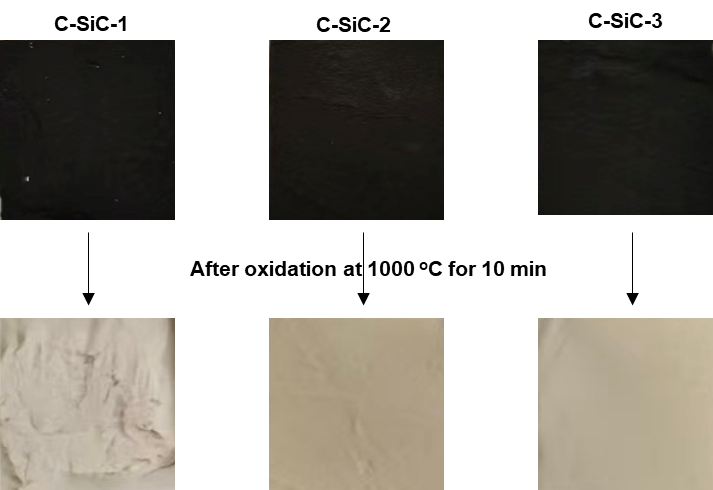
**

**Figure S36.** Photographs of the C-SiC fibrous aerogels before and after oxidation at 1000 ℃ for 10 min in air.

**
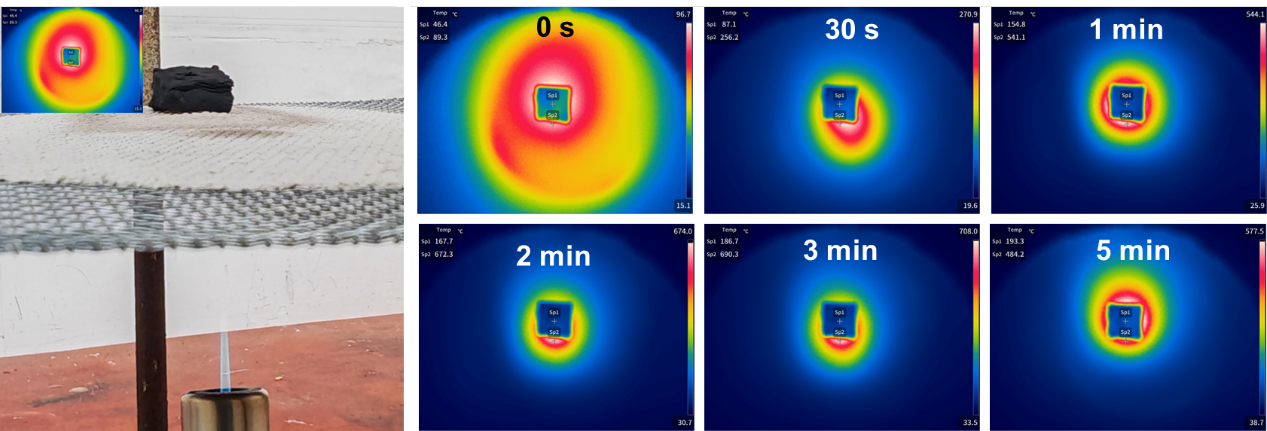
**

**Figure S37.** Optical and infrared images of the C-SiC fibrous aerogel being heated by a butane torch.

The N₂ adsorption isotherm of the C-SiC fibrous aerogel could be delineated into three distinct regions. First, at relative pressures (p/p₀) below 0.02, the N₂ adsorption capacity exhibited a rapid increase followed by swift saturation, indicative of the microporous nature of the fibers. In this ultra-low pressure range, the interaction between the microporous surfaces and N₂ molecules was intensified, leading to rapid adsorption until reaching a limit imposed by the micropore volume. Second, within the relative pressure range of 0.02 to 0.89, the adsorption curve demonstrated a linear ascent with a discernible inflection point corresponding to the transition from monolayer to multilayer adsorption. This behavior reflected the mesoporous characteristics of the fibers, attributable to the strong interactions between N₂ and the fibrous aerogel. Third, at relative pressures exceeding 0.89, the adsorption capacity increased sharply once more and approaches divergence, signifying the presence of macropores within the fibrous aerogel. As the partial pressure ratio rises, N₂ underwent pronounced liquefaction, and capillary condensation within the macropores becomes prominent. Nevertheless, complete pore filling was not achieved, resulting in the absence of a plateau in the adsorption isotherm. The C-SiC fibrous aerogels possessed a complex pore architecture, comprising features such as crack-like and wedge-shaped pores, which contributed to a broad pore size distribution and irregular pore morphology.

**Figure S38.** N₂ adsorption-desorption isotherms of the C-SiC fibrous aerogels.


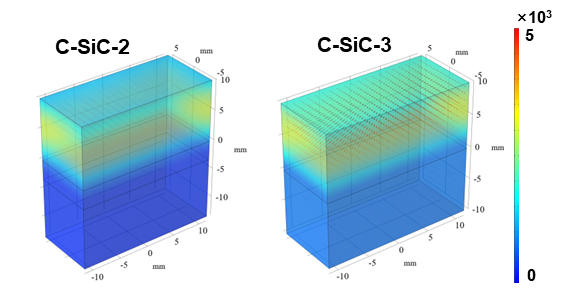


Figure S39. The simulation results of electric field distribution without and with C-SiC fibrous aerogel.

**Figure S40.** The electromagnetic shielding performance of C-SiC fibrous aerogels after being held in 1 M HCl for 7 days.

**Figure S41.** The electromagnetic shielding performance of C-SiC fibrous aerogels after being held in 6 M NaOH for 7 days.

**Table S1.** Elemental content changes at different stages during the preparation of the C-SiC-2 fibrous aerogels.

| **Temperature (^o^C)** | **Content (wt%)** | | | |
| --- | --- | --- | --- | --- |
|  | **Si** | **C** | **O** | **N** |
| 1300 | 27.05 | 44.23 | 18.64 | 10.08 |
| 1600 | 41.45 | 57.49 | 0.5 | 0.56 |
| 1800 | 37.90 | 61.47 | 0.31 | 0.32 |

**Table S2.** Elemental composition of the C-SiC fibrous aerogels obtained at 1800 ^o^C.

| **Sample name** | **Feeding ratio of polysiloxane to PAN** | **Si content**  **(wt%)** | **C content**  **(wt%)** | **C/Si**  **atomic ratio** | **O content**  **(wt%)** | **N content**  **(wt%)** |
| --- | --- | --- | --- | --- | --- | --- |
| C-SiC-1 | 0.5 | 19.43 | 79.83 | 9.59 | 0.32 | 0.42 |
| C-SiC-2 | 2.0 | 37.90 | 61.47 | 3.78 | 0.31 | 0.32 |
| C-SiC-3 | 3.0 | 40.64 | 58.70 | 3.37 | 0.32 | 0.34 |

**Table S3.** Grain sizes of the C-SiC fibrous aerogels.

| **Sample** | **1** | **2** | **3** |
| --- | --- | --- | --- |
| 1600 ℃ | 28 nm | 30 nm | 35 nm |
| 1800 ℃ | 39 nm | 39 nm | 46 nm |

**Table S4.** Statistical data of specific surface area, pore volume, and pore size in C-SiC fibrous aerogels calculated from the N₂ adsorption-desorption isotherm.

| **Sample name** | **Specific surface area (BET****)/****m^2^·g^-1^** | **Pore volume** **(NLDFT)/mL·g^-1^** | **Micropore volume (NLDFT)/mL·g^-1^** | | **Average pore diameter /nm (4V/A by BET)** | **Most Probable Pore Size /nm****(BJH Absorption)** |
| --- | --- | --- | --- | --- | --- | --- |
| C-SiC-1 | 287.65 | 0.35 | 0.12 | 4.9 | | 2.5 |
| C-SiC-2 | 373.23 | 0.40 | 0.16 | 4.6 | | 2.3 |
| C-SiC-3 | 228.76 | 0.29 | 0.10 | 5.4 | | 2.4 |

**Table S5.** Comparison of the C-SiC fibrous aerogel and different aerogels previously reported in literature.

| **Materials** | **Thermal conductivity**  **(25 ℃；****mW·m^-1^·K^-1^)** | **Ref.** |
| --- | --- | --- |
| ZrO_2_ sponge | 27 | [1] |
| SiO_2_-AlBSi aerogel | 25 | [2] |
| SiC nanowire aerogel | 26 | [3] |
| SiO_2_-Al_2_O_3_ sponge | 34 | [4] |
| SiC@SiO_2_ aerogel | 14 | [5] |
| Mullite aerogel | 22.8 | [6] |
| Mullite sponge | 30.7 | [7] |
| Zircon aerogel | 26 | [8] |
| Silica-Alumina aerogel | 29 | [9] |
| Aluminosilicate aerogel | 20 | [10] |
| SiO_2_-ZrO_2_ aerogel | 25.4 | [11] |
| SiO_2_-Al_2_O_3_ aerogel | 27 | [12] |
| BN/C/Al_2_O_3_ aerogel | 32 | [13] |
| SiO_2_-ZrO_2_ aerogel | 34 | [14] |
| Carbon Tube aerogel | 23 | [15] |
| **C-SiC fibrous** **aerogel** | **9.12** | **This work** |

**Table S6.** Related parameters of the electromagnetic shielding ability of the C-SiC fibrous aerogels.

| **Sample name** | **Thickness**  **(mm****)** | $\boldsymbol{\rho}$  **(mg·cm^-3^)** | **Average SE_T_ (dB)** | **Maximum SE (dB)** | **Minimum SE (dB)** | **SSE/t**  **(dB·cm^2^/g)** |
| --- | --- | --- | --- | --- | --- | --- |
| C-SiC-1 | 0.380 | 3.90 | 51.4 | 53.1 | 50.5 | 346,828.6 |
| C-SiC-2 | 3.131 | 3.41 | 22.7 | 23.2 | 22.5 | 21,261.2 |
| C-SiC-3 | 0.065 | 18.40 | 10.6 | 11.8 | 10.2 | 88,628.7 |

**Table S7.** Comparison of basic physical properties of the C-SiC fibrous aerogels.

| **Sample name** | **Specific surface area**  **(m^2^·g^-1^)** | **Free C**  **(wt%****)** | **Conductivity×****10^-6^**  **(S·m^-1^)** | **I_D_/I_G_** |
| --- | --- | --- | --- | --- |
| C-SiC-1 | 288 | 71.50 | 4.36 | 1.37 |
| C-SiC-2 | 373 | 45.23 | 1.85 | 1.23 |
| C-SiC-2 | 251 | 41.28 | 1.59 | 1.12 |

**Table S8.** Comparison of the density and intensity of the C-SiC fibrous aerogels in this work with those of other work reported in the literature.

| **Category** | **Material** | **SSE/t**  **(dB cm^2^g^-1^)** | **Density**  **(g·cm^-3^)** | **Ref.** |
| --- | --- | --- | --- | --- |
| Carbon Nanotube (CNT) based materials | Polyimide/CNT aerogels | 6470 | 0.018 | [16] |
|  | CNT sponges | 30444.4 | 0.01 | [17] |
|  | Aramid nanofiber/CNT aerogel | 33528.3 | 0.029 | [18] |
| MXene based materials | MXene/chitosan-derived hybrid carbon aerogel | 17184.8 | 0.0119 | [19] |
|  | MXene aerogels | 26 500 | 0.01 | [20] |
|  | Mxene/graphene porous film | 76422 | 0.41 | [21] |
|  | Ti_2_C_3_T_x_ MXene/single-walled carbon nanotube films | 15263.1 | 0.92 | [22] |
|  | MXene/CNTs/Aramid aerogels | 8060.7 | 0.0428 | [23] |
| Graphene based materials | Amide-connected rGO films | 5660 | 1.68 | [24] |
|  | Graphene aerogels | 173243 | 0.0037 | [25] |
| Graphite based materials | Polydopamine carbon aerogels | 40785.9 | 0.00311 | [26] |
|  | Wood-Derived anisotropic magnetic porous carbon | 881.9 | 0.288 | [27] |
|  | SiC-Coated carbonized natural loofah sponge | 421.4 | 0.637 | [28] |
| Metal based materials | Polysulfonamide/AgNWs aerogel | 14760.98 | 0.0123 | [29] |
|  | Ag-wrapped polyimide aerogel | 27090 | 0.11 | [30] |
|  | V_2_O_5_ nanowires-polyaniline aerogel | 2770.3 | 0.02 | [31] |
|  | **C-SiC fibrous** **aerogel** | **346828.6** | **0.0039** | **This work** |

**Movie S1.**

Infrared thermal imaging video of the C-SiC fibrous aerogel heated continuously by the butane flame

**Movie S2.**

Application demonstration of electromagnetic shielding and thermal insulation performance of the C-SiC fibrous aerogel

**References**

1. H. Wang, X. Zhang, N. Wang, et al., "Ultralight, scalable, and high-temperature-resilient ceramic nanofiber sponges*,"* *Science Advances* 3 e1603170. https://doi.org/10.1126/sciadv.1603170

2. Y. Si, X. Wang, L. Dou, J. Yu, B. Ding, "Ultralight and fire-resistant ceramic nanofibrous aerogels with temperature-invariant superelasticity*,"* *Science Advances* 4 eaas8925. https://doi.org/10.1126/sciadv.aas8925

3. L. Su, H. Wang, M. Niu, et al., "Ultralight, recoverable, and high-temperature-resistant SiC nanowire aerogel*,"* *ACS Nano* 12 (2018): 3103-3111. https://doi.org/10.1021/acsnano.7b08577

4. C. Jia, L. Li, Y. Liu, et al., "Highly compressible and anisotropic lamellar ceramic sponges with superior thermal insulation and acoustic absorption performances*,"* *Nature Communications* 11 (2020): 3732. https://doi.org/10.1038/s41467-020-17533-6

5. L. Su, H. Wang, M. Niu, et al., "Anisotropic and hierarchical SiC@SiO_2_ nanowire aerogel with exceptional stiffness and stability for thermal superinsulation*,"* *Science Advances* 6 (2020): eaay6689. https://doi.org/10.1126/sciadv.aay6689

6. X. Cheng, Y.-T. Liu, Y. Si, J. Yu, B. Ding, "Direct synthesis of highly stretchable ceramic nanofibrous aerogels via 3D reaction electrospinning*,"* *Nature Communications* 13 (2022): 2637. https://doi.org/10.1038/s41467-022-30435-z

7. L. Li, C. Jia, Y. Liu, et al., "Nanograin-glass dual-phasic, elasto-flexible, fatigue-tolerant, and heat-insulating ceramic sponges at large scales*,"* *Materials Today* 54 (2022): 72-82. https://doi.org/10.1016/j.mattod.2022.02.007

8. J. Guo, S. Fu, Y. Deng, et al., "Hypocrystalline ceramic aerogels for thermal insulation at extreme conditions*,"* *Nature* 606 (2022): 909-916. https://doi.org/10.1038/s41586-022-04784-0

9. Y. Cheng, B. Ma, P. Hu, et al., "Flexible and transformable ceramic aerogels via a fire-reborn strategy for thermal superinsulation in extreme conditions*,"* *Advanced Functional Materials* 33 (2023): 2309148. https://doi.org/10.1002/adfm.202309148

10. L. Li, Y. Zhou, Y. Gao, et al., "Large-scale assembly of isotropic nanofiber aerogels based on columnar-equiaxed crystal transition*,"* *Nature Communications* 14 (2023): 5410. https://doi.org/10.1038/s41467-023-41087-y

11. S. Dang, J. Guo, Y. Deng, et al., "Highly-buckled nanofibrous ceramic aerogels with ultra-large stretchability and tensile-insensitive thermal insulation*,"* *Advanced Materials* 37 (2025): 2415159. https://doi.org/10.1002/adma.202415159

12. Z. Xu, Y. Liu, Q. Xin, et al., "Ceramic meta-aerogel with thermal superinsulation up to 1700 °C constructed by self-crosslinked nanofibrous network via reaction electrospinning*,"* *Advanced Materials* 36 (2024): 2401299. https://doi.org/10.1002/adma.202401299

13. H. Liu, X. Zhang, Y. Liao, et al., "Building-envelope-inspired, thermomechanically robust all-fiber ceramic meta-aerogel for temperature-controlled dominant infrared camouflage*,"* *Advanced Materials* 36 (2024): 2313720. https://doi.org/10.1002/adma.202313720

14. T. Zhou, L. He, Y. Zhen, et al., "Superstrong lightweight aerogel with supercontinuous layer by surface reaction*,"* *Advanced Materials* 37 (2025): 2418083.

https://doi.org/10.1002/adma.202418083

15. H.-J. Zhan, K.-J. Wu, Y.-L. Hu, et al., "Biomimetic carbon tube aerogel enables super-elasticity and thermal insulation*,"* *Chem* 5 (2019): 1871-1882.

https://doi.org/10.1016/j.chempr.2019.04.025

16. S. Qiao, H. Chen, Y. Zhao, Z. Wang, J. Yan, "Bioinspired polyimide/carbon nanotube aerogels with core-radiating and omasum-like morphology toward excellent electromagnetic shielding and superior elasticity*,"* *Advanced Materials* 37 (2025): e13423.

https://doi.org/10.1002/adma.202513423

17. D. Lu, Z. Mo, B. Liang, et al., "Flexible, lightweight carbon nanotube sponges and composites for high-performance electromagnetic interference shielding*,"* *Carbon* 133 (2018): 457-463. https://doi.org/10.1016/j.carbon.2018.03.061

18. P. Hu, J. Lyu, C. Fu, et al., "Multifunctional aramid nanofiber/carbon nanotube hybrid aerogel films*,"* *ACS Nano* 14 (2020): 688-697. https://doi.org/10.1021/acsnano.9b07459

19. S. Wu, D. Chen, W. Han, et al., "Ultralight and hydrophobic MXene/chitosan-derived hybrid carbon aerogel with hierarchical pore structure for durable electromagnetic interference shielding and thermal insulation*,"* *Chemical Engineering Journal* 446 (2022): 137093. https://doi.org/10.1016/j.cej.2022.137093

20. N. Wu, Y. Yang, C. Wang, et al., "Ultrathin cellulose nanofiber assisted ambient-pressure-dried, ultralight, mechanically robust, multifunctional mxene aerogels*,"* *Advanced Materials* 35 (2023): 2207969. https://doi.org/10.1002/adma.202207969

21. X. Tang, J. Luo, Z. Hu, et al., "Ultrathin, flexible, and oxidation-resistant MXene/graphene porous films for efficient electromagnetic interference shielding*,"* *Nano Research* 16 (2023): 1755-1763. https://doi.org/10.1007/s12274-022-4841-1

22. B. Zhou, Y. Li, Z. Li, et al., "Fire/heat-resistant, anti-corrosion and folding Ti_2_C_3_T_x_ mxene/single-walled carbon nanotube films for extreme-environmental EMI shielding and solar-thermal conversion applications*,"* *Journal of Materials Chemistry C* 9 (2021): 10425-10434. https://doi.org/10.1039/D1TC00289A

23. Z. Yan, Y. Ding, M. Huang, et al., "MXene/CNTs/aramid aerogels for electromagnetic interference shielding and joule heating*,"* *ACS Applied Nano Materials* 6 (2023): 6141-6150. https://doi.org/10.1021/acsanm.3c00249

24. J. Zhang, T. Xu, L. Ding, et al., "Bridging graphene for films with superior mechanical and electrical performance for electromagnetic interference shielding*,"* *eScience* 5 (2025): 100407. https://doi.org/10.1016/j.esci.2025.100407

25. E. Zhu, K. Pang, Y. Chen, et al., "Ultra-stable graphene aerogels for electromagnetic interference shielding*,"* *Science China Materials* 66 (2023): 1106-1113. https://doi.org/10.1007/s40843-022-2208-x

26. P. Chen, S. He, T. Wang, et al., "Melanin-like nanofibers with highly ordered structures achieve ultrahigh specific electromagnetic interference shielding efficiency*,"* *Nature Communications* 16 (2025): 7127. https://doi.org/10.1038/s41467-025-62367-9

27. Y. Zheng, Y. Song, T. Gao, et al., "Lightweight and hydrophobic three-dimensional wood-derived anisotropic magnetic porous carbon for highly efficient electromagnetic interference shielding*,"* *ACS Applied Materials & Interfaces* 12 (2020): 40802-40814.

https://doi.org/10.1021/acsami.0c11530

28. S. Li, D. Liu, W. Li, G. Sui, "Strong and heat-resistant SiC-coated carbonized natural loofah sponge for electromagnetic interference shielding*,"* *ACS Sustainable Chemistry & Engineering* 8 (2020): 435-444. https://doi.org/10.1021/acssuschemeng.9b05723

29. J. Wang, Q. Zhang, S. Zhang, et al., "Multifunctional polysulfonamide/AgNWs aerogel for electromagnetic interference shielding*,"* *Composites Communications* 56 (2025): 102346. https://doi.org/10.1016/j.coco.2025.102346

30. T. Xue, Z. Fu, D. Yu, et al., "Lightweight and flexible Ag-wrapped polyimide aerogel fabrics for electromagnetic interference shielding*,"* *Composites Communications* 43 (2023): 101732. https://doi.org/10.1016/j.coco.2023.101732

31. A. Puthiyedath Narayanan, K. N. Narayanan Unni, K. Peethambharan Surendran, "Aerogels of V_2_O_5_ nanowires reinforced by polyaniline for electromagnetic interference shielding*,"* *Chemical Engineering Journal* 408 (2021): 127239. https://doi.org/10.1016/j.cej.2020.127239
